# Sterile sentinels and MinION sequencing capture active soil microbial communities that differentiate crop rotations

**DOI:** 10.1186/s40793-024-00571-8

**Published:** 2024-05-07

**Authors:** Sonya R. Erlandson, Patrick M. Ewing, Shannon L. Osborne, R. Michael Lehman

**Affiliations:** 1 USDA-ARS-North Central Agricultural Research Laboratory, Brookings, SD 57006 USA; 2 USDA-ARS-Food Systems Research Unit, Burlington, VT 57006 USA

**Keywords:** Oxford nanopore minion, crop rotation, Ingrowth bags, active microbial community, Soil microbiome, Microbial community monitoring

## Abstract

**Background:**

Soil microbial communities are difficult to measure and critical to soil processes. The bulk soil microbiome is highly diverse and spatially heterogeneous, which can make it difficult to detect and monitor the responses of microbial communities to differences or changes in management, such as different crop rotations in agricultural research. Sampling a subset of actively growing microbes should promote monitoring how soil microbial communities respond to management by reducing the variation contributed by high microbial spatial and temporal heterogeneity and less active microbes. We tested an in-growth bag method using sterilized soil in root-excluding mesh, “sterile sentinels,” for the capacity to differentiate between crop rotations. We assessed the utility of different incubation times and compared colonized sentinels to concurrently sampled bulk soils for the statistical power to differentiate microbial community composition in low and high diversity crop rotations. We paired this method with Oxford Nanopore MinION sequencing to assess sterile sentinels as a standardized, fast turn-around monitoring method.

**Results:**

Compared to bulk soil, sentinels provided greater statistical power to distinguish between crop rotations for bacterial communities and equivalent power for fungal communities. The incubation time did not affect the statistical power to detect treatment differences in community composition, although longer incubation time increased total biomass. Bulk and sentinel soil samples contained shared and unique microbial taxa that were differentially abundant between crop rotations.

**Conclusions:**

Overall, compared to bulk soils, the sentinels captured taxa with copiotrophic or ruderal traits, and plant-associated taxa. The sentinels show promise as a sensitive, scalable method to monitor soil microbial communities and provide information complementary to traditional soil sampling.

**Supplementary Information:**

The online version contains supplementary material available at 10.1186/s40793-024-00571-8.

## Background

Soil microbial communities exhibit high spatial and temporal heterogeneity [9, 10, 30]. Microbial community spatial heterogeneity arises from high spatial turnover across multiple scales and, critically, strong spatial heterogeneity can dampen the ability to detect responses to variables of interest [9, 10, 14, 30, 41, 44]. In regard to temporal heterogeneity, microbial biomass turns over on an order of weeks to months depending on taxonomic group [48, 51]. Additionally, relic DNA can contribute to obscuring important differences in microbial communities [9, 70]. In short, measuring microbial communities to detect their responses to specific changes in management across space and time, for example across agricultural fields and growing seasons, is difficult. These challenges in assessing microbial communities are relevant to the wide interest in monitoring microbes, for example, as a soil health indicator in agricultural systems [21, 58]. Spatial and temporal heterogeneity and relic DNA may obscure important differences in microbial communities and thus impede such monitoring applications.

Although there are many ways to measure microbial communities, bulk soil DNA sequencing has become a standard approach. DNA sequencing is relatively fast and easy, but there are challenges associated with using bulk soil DNA profiles as a soil health metric. These include the high diversity and heterogeneity that make community data difficult to interpret, a disconnect between structure and function, and collateral sampling of inactive microbes [7, 21, 58]. This limits the utility of bulk soil DNA profiling methods for assessing microbial responses to management, inferring microbial impacts on soil quality and plant health, and ultimately for selecting management interventions based on microbial communities. Other methods, including utilizing RNA or chemically excluding relic DNA, have also been used, with their own drawbacks [9, 70, 71].

A complementary method is to selectively sample an actively growing microbial community. This may reduce undesirable heterogeneity to improve detection of changes in microbial communities in response to changes in the soil environment, including from agricultural management such as crop rotation. Primarily sampling the DNA from actively growing microbes should reflect the current soil environment and reduce the contributions from less active taxa and dormant cells in bulk soil. Just 0.2-5% of microbial biomass in soil is estimated to be active at any moment, and only 60% is potentially active [7]. Moreover, the transition from dormant to active takes hours to days [7]. Ingrowth cores or bags are one approach for sampling actively growing organisms [2, 47, 63]. Typically, mesh bags filled with sterilized sand or soil are incubated in the soil, allowing growing organisms small enough to cross the mesh barrier to grow into the sterile substrate. The ingrowth bags then are retrieved after an incubation period. This approach has been used to estimate the production, biomass turnover, or composition of fine roots, mycorrhizal fungal dynamics, and bacterial dispersal [3, 11, 18, 22, 61, 64, 69]. Ingrowth bags have not been well-assessed as a monitoring tool for soil microbial communities and the limitations of using ingrowth bags versus bulk soil have not been evaluated using MinION sequencing. We expected that using sterilized soil in root-excluding mesh bags could help capture an active subset of the soil microbial community that is more relevant to management differences than communities in bulk soil.

We tested the power of sterile soil traps, dubbed “sterile sentinels,” to differentiate soil microbial communities under corn grown within low (2 crops) and high (4 crops) diversity annual crop rotations. Sterile sentinels consisted of root-excluding mesh bags containing autoclaved soil as barren substrata for colonization. We buried sterile sentinels in the crop row in late June, incubated sentinels for 1, 2, 4, 8, or 12 weeks before retrieval, and sampled bulk soils concurrently with sentinel placement and retrieval. The experiment was conducted for two consecutive growing seasons under the corn phase for both crop rotations. Bacterial and fungal metabarcoding was performed on an Oxford Nanopore MinION. We sought to answer three questions: (1) Do sterile sentinels capture microbial communities that differentiate crop rotations better than bulk soil? (2) What incubation time best differentiates crop rotation microbial communities? (3) Which microbes are associated with sample type (sentinels versus bulk soil) and with each crop rotation within sample type?

We selected two annual crop rotations which differed in crop rotational diversity because we expected to see significant compositional differences in the soil microbial communities based on prior analyses of rhizosphere communities [4]. We hypothesized that (1) sterile sentinels would capture a subset of the microbial community with greater statistical power to differentiate between crop rotations compared to bulk soil, due to sentinels capturing an actively growing subset of the bulk soil. We hypothesized that (2) sentinels incubated in soil from early to mid-growing season would best differentiate between crop rotations. We reasoned that with longer incubation time the current corn crop influence would homogenize the active community and colonization by more taxa would increase sampling noise making it harder to distinguish rotations. Finally, we hypothesized that (3) sterile sentinels would be colonized by actively growing taxa with high dispersal ability and the ability to exploit new resources quickly, in short, copiotroph, or ruderal taxa. Sentinels were expected to contain fewer taxa than bulk soil. We also expected the low diversity rotation to have a higher relative abundance of species that can be classified as copiotrophs or ruderals, and pathogens, and a lower relative abundance of potentially beneficial taxa relative to the high diversity rotation. We previously observed more pathogens in low diversity rotations in this field experiment and corn yields are lower in low diversity rotations compared to more diverse rotations, which was partly due to microbial community differences [4].

## Methods

### Research plots

A long-term research experiment was established in 2000 at the Eastern South Dakota Soil and Water Research Farm in Brookings, South Dakota, USA (44°21’ N; 96°48’ W) to compare a two-year corn (*Zea mays* L.)-soybean (*Glycine max* L. Merr.) (CS) crop rotation with more diversified crop rotations, including a four-year corn-soybean-spring wheat (*Triticum aestivum* L.)-pea (*Pisum sativum* subsp. *arvense* L. Asch.) (CSSwP) crop rotation. At this site, elevation is 500 m, the thirty-year mean annual precipitation is 580 mm, and mean annual temperature is 6.2 °C. The Mollisol soils are a moderately drained, Barnes sandy clay loam with organic carbon content of 18 g C kg^–1^ soil (0–15 cm). Crop rotation treatments were established in a randomized complete block design with four replications; each crop in a rotation sequence is present each year. The plots (93 m^2^) were no-till with 85% nitrogen fertilization based on locally recommended rates according to fall soil tests and crop yield goals (for 2020: corn = 7.84 Mg ha^− 1^, spring wheat = 2.95 Mg ha^− 1^; for 2021: corn = 9.4 Mg ha^− 1^, spring wheat = 3.36 Mg ha^− 1^); corn received 15 kg N ha^− 1^ and spring wheat received 15 kg N ha^− 1^ and 12 kg K_2_O ha^− 1^ at planting. All plots received herbicide-based weed management as needed. For each year of sampling, plots in the corn phase of the CS and CSSwP rotations were studied.

### Sterile sentinel construction

Sterile sentinels were constructed of 31 μm nylon mesh to inhibit root access; 5 cm x 5 cm bags were created with heat-sealing tape and filled with 12 g of soil that was autoclaved twice for 40 min at 121 °C with a 24-hour rest period between cycles. Sterile sentinels were freshly prepared each year, including autoclaving the soil. Soil to fill sterile sentinels was collected once from the top 10 cm of border strips within an adjacent field experiment with the same soil type which had been cropped with corn, soybean, and small grains under no-till over the previous 20 years. This soil was air-dried, sieved (2 mm), and mixed before autoclaving.

### Field placement and sampling

**Fig. 1:**
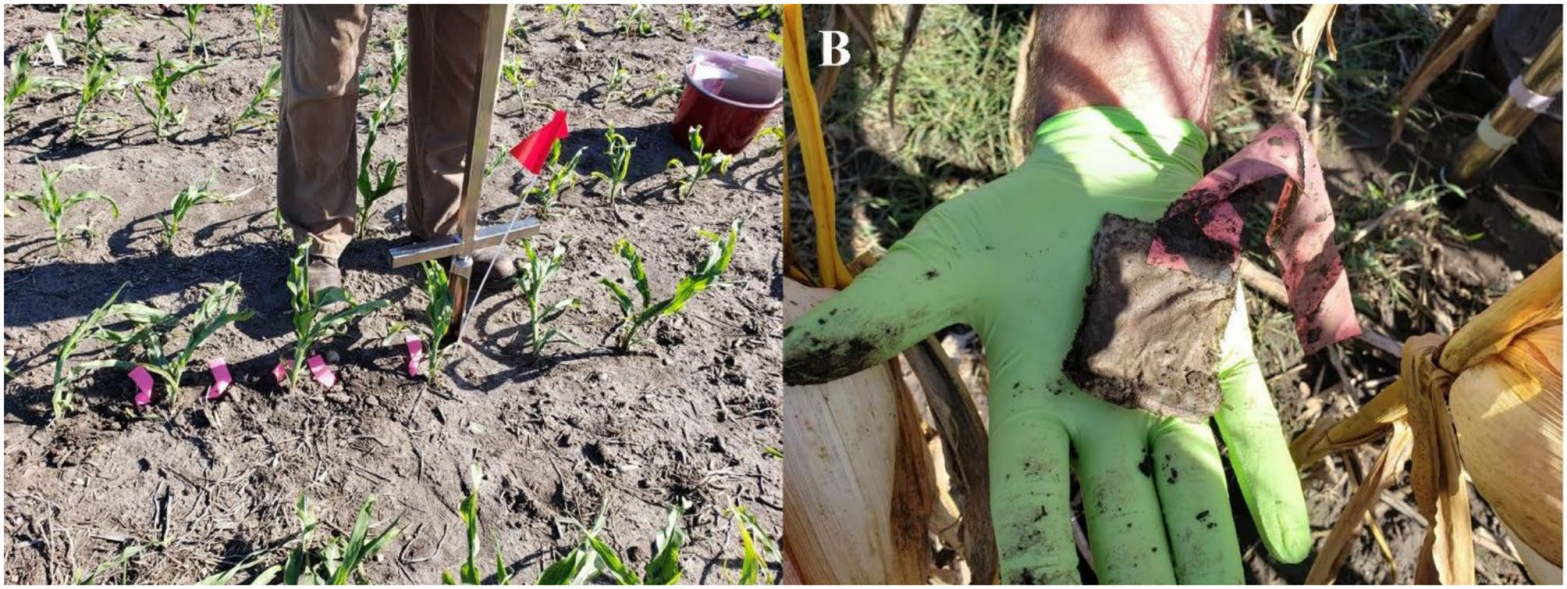
(**A**) Sampling bulk soil at week 0, late June. Pink flagging tape marks sterile sentinel locations. Sterile sentinels were buried just prior to bulk soil sampling seen in photo. Each plot had six sets of five sentinels. (**B**) A retrieved sentinel from week 12, mid-September

In 2020 and 2021, six sets of five sterile sentinels were buried vertically with their midpoint at 5 cm, in the corn row, after corn fertilization (corn stage V4-V6, late June, Fig. 1A). Three sentinel sets were placed two corn rows in from the plot edge; the remaining three sets were placed two rows north from the plot center corn row. Sentinels were collected at 1, 2, 4, 8, and 12 weeks after placement corresponding to late June, early July, late July, mid-August, and mid-September (Fig. 1B).

This design resulted in six sentinel traps for each sampling week per plot, replicated by four plots per rotation treatment (CS and CSSwP), and 240 total traps were placed each year. At sentinel placement and each sampling week, a 3.2 cm diameter bulk soil core was taken adjacent to the collected sentinel. The soil core was cut to collect only the 2.5–7.5 cm depth to correspond with the sterile sentinel environment. The six sentinels were composited to yield one sentinel sample per plot for each sampling week; the six bulk soil cores were composited the same way. Soils were stored in a cooler on ice until processing within 4 h. Composited samples were passed through a 2 mm sieve, which was sanitized with 70% ethanol between samples, and an approximate 20 g sub-sample was frozen at -80 °C for DNA extraction. A 10 g subsample was weighed, oven-dried at 105 °C for 24 h, then weighed again to determine gravimetric soil moisture content.

### Molecular work

DNA was extracted in triplicate with DNeasy PowerSoil Pro (Qiagen, USA). Total extracted DNA was quantified using the Invitrogen Quant-iT dsDNA Broad Range Assay Kit (Thermo Fischer Scientific, USA), then triplicate extractions were combined prior to PCR. A two-step PCR was performed; an amplicon specific PCR followed by a PCR to attach barcodes. All PCR was done with Phusion Green Hot Start II High-Fidelity PCR master mix. Amplicon PCR (PCR1) was done in triplicate to amplify the full-length 16 S rRNA gene with bacterial primers 27 F and 1492R [29, 59] and the partial 18 S–5.8 S-28 S rRNA gene with Eukaryote primers SSU515fngs and TW13 [54, 56, 59]; both had Oxford Nanopore specific adapters attached. Technical replicates were visualized on agarose gel, combined by equal volume, cleaned with 1:1 AMPure XP beads (Beckman Coulter Life Sciences, USA), and quantified with a Quant-it High Sensitivity or Broad Range kit (Thermo Fischer Scientific, USA). Barcode PCR (PCR2) was done with the Oxford Nanopore PCR Barcoding Expansion 1–96 Kit (EXP-PBC096, Oxford Nanopore Technologies Ltd, UK), following the recommended protocol. Libraries were prepared and sequenced following the Oxford Nanopore EXP-PBC096 protocol using the Oxford Nanopore Ligation Sequencing Kit (SQK-LSK109, Oxford Nanopore Technologies Ltd, UK) for flow cell v9.4 on a MinION Mk1C device. Control samples for the experimental set-up, DNA extraction, and PCR were also included in the library preparation and sequencing: autoclaved soil, DNA extraction blank, a mock community positive control, PCR1 and PCR2 negative controls. The ZymoBIOMICS Microbial DNA Community Standard (#D6305, Zymo Research, USA) was the mock community positive control. One flow cell was used for each library (bacteria 2020, bacteria 2021, eukaryotes 2020, eukaryotes 2021).

### Bioinformatics

MinION fast5 files were base called and demultiplexed with guppy v4.2.2. Read quality and filtering parameters were selected by examining plots generated by NanoPlot v1.31.0 [17]. Demultiplexed reads were quality filtered with NanoFilt v2.7.1 with minimum average read quality score of 8 and read length between 200 and 3500 base pairs for eukaryote reads; for bacteria the quality filtering parameters were minimum average read quality score of 12 and a read length between 1000 and 1700 base pairs [17]. Quality filtered reads were trimmed to remove any remaining adapter and primer sequences with cutadapt v3.2 [39].

Popular bioinformatics workflows for generating operational taxonomic units or amplicon sequence variants were not applicable to MinION sequencing data due to differences in error rates, so we tested two approaches to grouping reads and assigning taxonomy. The first approach grouped reads and assigned taxonomy by aligning each read to a reference database with minimap2 v2.22, which was designed to align reads with high error rates to references [32, 33]. We used minimap2 to align reads to the reference database using the default settings for Oxford Nanopore reads. Aligned reads were filtered to exclude alignments with a quality score less than 4 and sequence divergence from a reference greater than 0.1. If a read was aligned to more than one reference sequence, only the alignment designated as primary by minimap2 (arbitrarily) was retained. The number of reads that aligned to the same reference sequence were counted to construct a count table. The SILVA reference database SSUv138 was used for bacteria and UNITE version 9 eukaryotes dynamic 29.11.2022 for eukaryotes [1, 49].

The second approach used Emu, which is built on minimap2. Emu uses read mapping alignment probabilities and an expectation-maximization algorithm to estimate taxonomic composition at the species level for Oxford Nanopore reads [15]. An Emu-provided database was used for bacterial reads. It consisted of the rrnDB v5.6 and NCBI 16 S rRNA gene RefSeq databases downloaded on September 17, 2020; containing 49,301 sequences from 17,555 unique bacterial and archaeal taxa. The UNITE version 9 all eukaryotes dynamic 29.11.2022 database was used for eukaryotes. We evaluated the two methods based on the similarity of the realized mock community to the theoretical mock community composition (i.e., similarity of the total number of taxa and proportion of each taxon) and the total number of reads retained (Supplementary Figs. 1 and 2). We chose to use Emu for bacteria because the number and proportions of bacterial taxa in the Emu mock community more closely matched the theoretical mock community than the minimap2 mock community (Supplementary Fig. 1). The realized mock communities for Emu and minimap2 were similar for eukaryotes, but minimap2 retained more reads, therefore we used minimap2 for eukaryotes (Supplementary Fig. 2). The eukaryote datasets were filtered to reads that mapped to kingdom Fungi to make bulk soil and sentinel samples more comparable, since sentinel size exclusion would affect larger eukaryotes, such as earthworms. Both bacterial and fungal datasets were filtered to exclude taxa represented by fewer than three reads.

Bacterial traits, including genome size, growth rate, and motility, were assigned at the species level by matching NCBI taxonomy ids from Emu taxonomy and the bacteria traits data product collected by Madin et al. [38]. The phenotypic and genomic traits for bacteria and archaea were aggregated from 26 curated, reliable sources, see Madin et al. [38] for a complete list of datasets and traits from each data set. Bacterial pathogens were determined at the genus level with FAPROTAX [36] and manual curation was done at the species level to ensure correct pathogen assignment using Bull et al. [8] as a reference. Fungal guilds were assigned by genus name from the FunGuild database [42].

### Statistical analysis

All statistical analyses were completed in R v4.1.1 [50] and data visualized with the R package ggplot2 [65]. Sampling weeks and year were analyzed individually, except richness t-tests, redundancy analysis and *R*^2^ variance paired t-tests. Sentinel and bulk soil samples from the same plot were paired for t-tests where applicable.

Basic community statistics were examined for each dataset. Rarefaction curves were generated to examine the effect of sampling depth on richness. Hill numbers for *q* = 0 and *q* = 1 were calculated on rarefied data tables using vegan and hillR packages and were compared between sample types and crop rotations [12]; Chiu & Chao [13, 31, 45]. Hill numbers are the effective number of species in an assemblage. The term *q* defines the sensitivity of a diversity to species frequency. Hill numbers are equivalent to species richness when *q* = 0 and the exponent of Shannon entropy when *q* = 1, and the units are always species [12]. Count tables were rarefied to the lowest sample size, as follows: bacteria 2020, 8745; bacteria 2021, 4168; fungi 2020, 1888; fungi 2021, 1062.

Redundancy analysis (RDA) tested relative explanatory power of sample type, sampling week, and crop rotation for microbial community composition; community data tables were center log ratio transformed and RDA performed using vegan [45].

To test the statistical power of sentinels versus bulk soil to differentiate between CS and CSSwP microbial communities we used permANOVA (adonis2) in vegan [45] and paired t-tests. Sample by taxa matrices were center log ratio transformed using CLR in vegan. PermANOVAs with rotation as the predictor of community distance (Euclidean) were done for each sampling week and type. A two-tailed, paired t-test of permANOVA *R*^2^ values was used to determine the statistical power of sentinel versus bulk soil for discriminating microbial community differences between crop rotations. To test if incubation time was important to distinguishing crop rotation microbial communities, sampling week (incubation time) was tested as a predictor of the permANOVA *R*^2^ in a model with amplicon, sample type (bulk or sentinel), and all interactions as predictors. Sampling week was tested as a linear and second-order polynomial.

To test if traits or pathogens were more abundant in sample type or crop rotation, differential abundance was analyzed for the sample by trait matrices for fungal trophic guild and bacterial motility type. Bacterial doubling time and genome size were tested between sample type and rotation for each sample type with Student’s t-tests or Wilcoxon t-tests. Differentially abundant species were identified for bacteria and fungi to determine taxa associated with sample type and crop rotation. Differential abundance was analyzed using the t-test in ALDEx2 [23]. Taxa or functional groups were considered differentially abundant at an absolute effect size greater than 1 (ALDEx2 recommendation). In ALDEx2, the effect size is the median of the ratio of the between-group difference and the larger of the variances within group [23]. Differential abundance t-tests were done between sentinel and bulk soil pairs from the same plot (*n* = 8) and between crop rotation for sentinels and bulk soil separately, for each week (*n* = 4).

## Results

### Microbial biomass, diversity, and composition

**Fig. 2:**
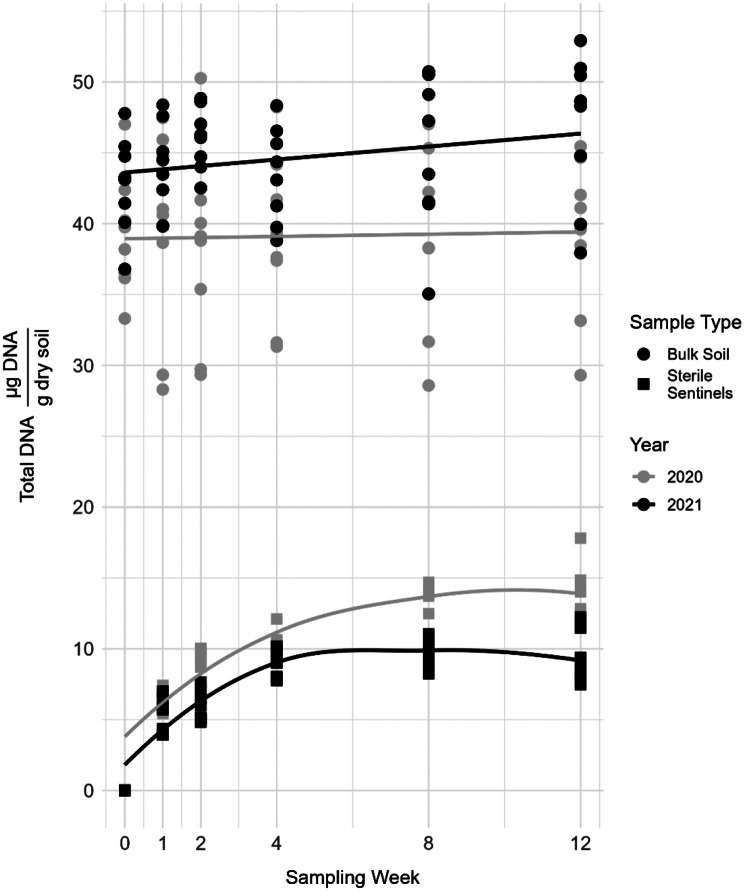
Total DNA extracted from sentinel and bulk soil samples, expressed on a dry soil mass basis. Bulk soil contained at least twice as much DNA as sterile sentinels. Total DNA extracted from sentinels increased until week 8 when it leveled off in both 2020 and 2021; sentinel data are shown with a loess model fit with geom_smooth in ggpot2. Total DNA extracted from bulk soils was similar across sampling weeks in both years; bulk data are shown with a linear model fit with geom_smooth in ggplot2. The sterile sentinel week 0 points represent the DNA extracted from the autoclaved soil control samples in 2020 and 2021

Total DNA (µg DNA/gram dry soil) extracted increased in sterile sentinels from week 1–8 and plateaued at weeks 8–12 in both 2020 and 2021 while total DNA remained high and constant from bulk soils (Fig. 2). The total DNA extracted from autoclaved soils was near zero (represented by week 0 sterile sentinel points in Fig. 2).

As a proxy for total biomass, this suggests that sentinels were colonized by actively growing microbes, had much lower total biomass than the bulk soil, and stopped accumulating biomass between weeks 8–12 (mid-August and mid-September). Autoclaved soil used for filling sterile sentinels contained very few sequences from *Bacillaceae* or *Spizellomycetaceae* and these specific reads were not detected in other samples.

As few studies have used Oxford Nanopore sequencing or a selective technique like sterile sentinels for metabarcoding of soil microbial communities, we present detailed sequencing and community results. The mean total number of reads across the four MinION flow cells was 3.2 million. The mean number of reads retained for all libraries after quality control was 807,364 with a mean of 1478 unique taxa. Read count summaries for each library at different bioinformatics steps are listed in Supplementary Table 1. Rarefaction curves reached a plateau for bacteria but did not reach a plateau for fungi (Supplementary Fig. 3). The method of read grouping likely influenced the shape of the rarefaction curves. The error correction in Emu collapses sequencing reads into fewer operational taxonomic units, whereas minimap2 alone generates more rare taxa. It is likely that greater sequencing depth would have recovered more bacterial and fungal taxa.

Bacterial richness and diversity were higher in sentinels than bulk soils in 2021, but in 2020 there was no statistical difference in richness and diversity (t-test including all time points, *p* = 0.05, Supplementary Table 2). Bulk soils had higher fungal richness and diversity than sterile sentinels (t-test including all time points, *p* = 0.001, Supplementary Table 2). Richness and diversity were significantly higher in the CS rotation for 2021 fungal bulk soils (t-test, *p* = 0.005). Bacterial diversity was marginally higher in the CSSwP rotation in 2021 (t-test, *p* = 0.04). Richness and diversity did not differ between crop rotations for bacteria or fungi in other years or sample types (t-test including all time points, *p* > 0.05, Supplementary Figs. 3–4).

**Fig. 3:**
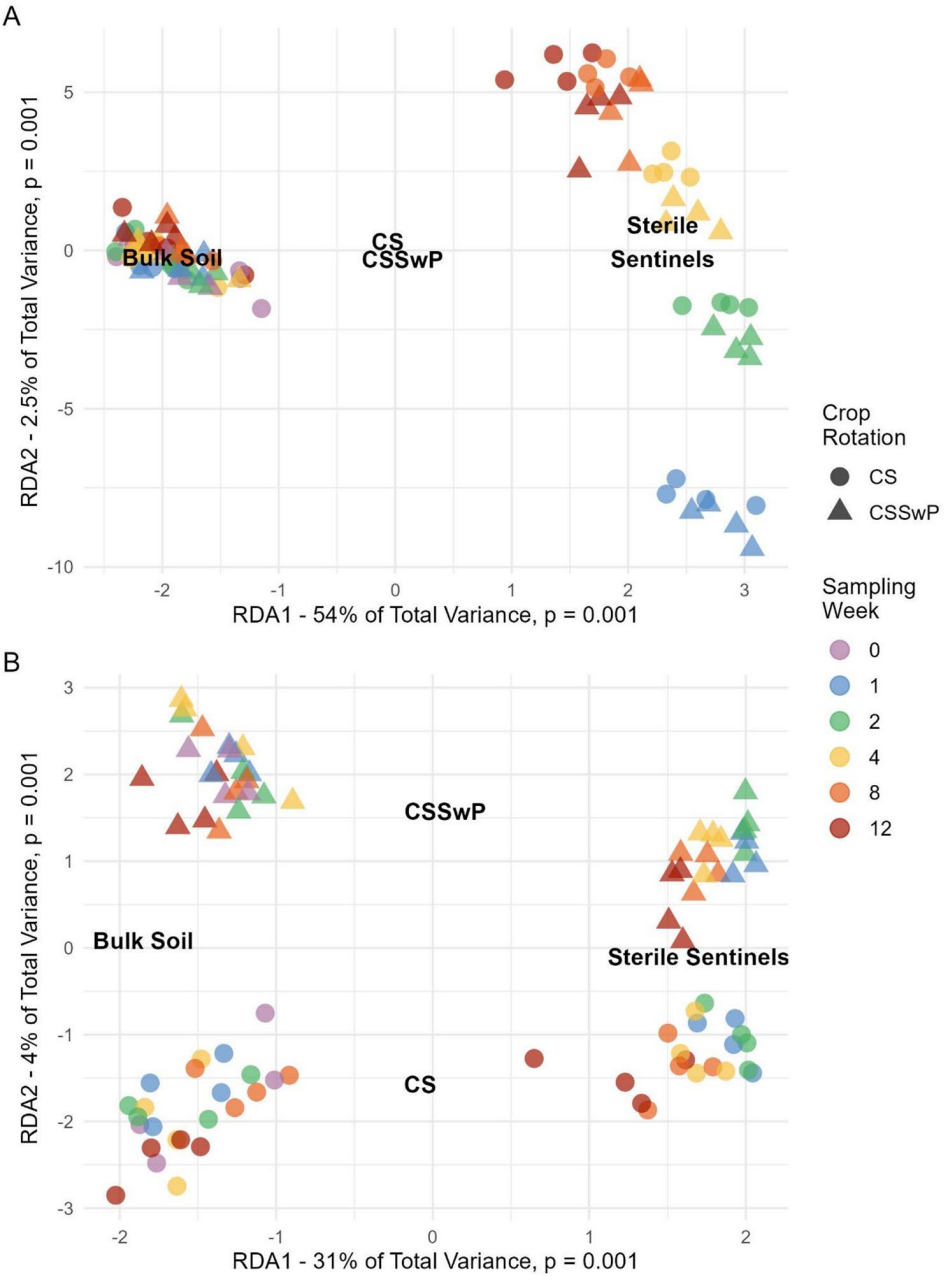
Redundancy analysis ordinations of bacteria 2020 (**A**) and fungi 2020 datasets (**B**) with sample type, sampling week and crop rotation as predictors. Colored points represent individual samples. The amount of total variance explained by each RDA axis is indicated along with significance. Ordinations are scaled to show relationship between samples (scaling = 2). Text indicates the centroids for categorical predictors (sample type and crop rotation). The RDA ordinations reveal differences in predictor hierarchy. Variance in bacterial community composition is best explained by sample type, followed by sampling week, and finally crop rotation. Fungal composition difference is best captured by sample type and crop rotation, then sampling week. Results for 2021 datasets were very similar and these data are shown in Supplementary Fig. 6. Summaries of all RDA statistics are in Supplementary Table 3

Redundancy analysis (RDA) of each dataset revealed differences in predictor relative importance for bacteria and fungi. Bacterial community composition was best explained by sample type (2020 *p* = 0.001, 2021 *p* = 0.001) followed by sampling week (2020 *p* = 0.001, 2021 *p* = 0.001) and crop rotation (2020 *p* = 0.056, 2021 *p* = 0.001), and the importance of sampling week was strongly driven by sterile sentinels (Fig. 3A, Supplementary Fig. 6A, Supplementary Table 3). Fungal community composition was best described by sample type (2020 *p* = 0.001, 2021 *p* = 0.001), then crop rotation (2020 *p* = 0.001, 2021 *p* = 0.001) and sampling week (2020 *p* = 0.001, 2021 *p* = 0.001) (Fig. 3B, Supplementary Fig. 6B, Supplementary Table 3). RDA results were the same in 2020 (Fig. 3A-B, Supplementary Table 3) and 2021 (Supplementary Fig. 6A-B, Supplementary Table 3).

### Crop rotation microbial community differentiation in sentinels versus bulk soil

**Fig. 4:**
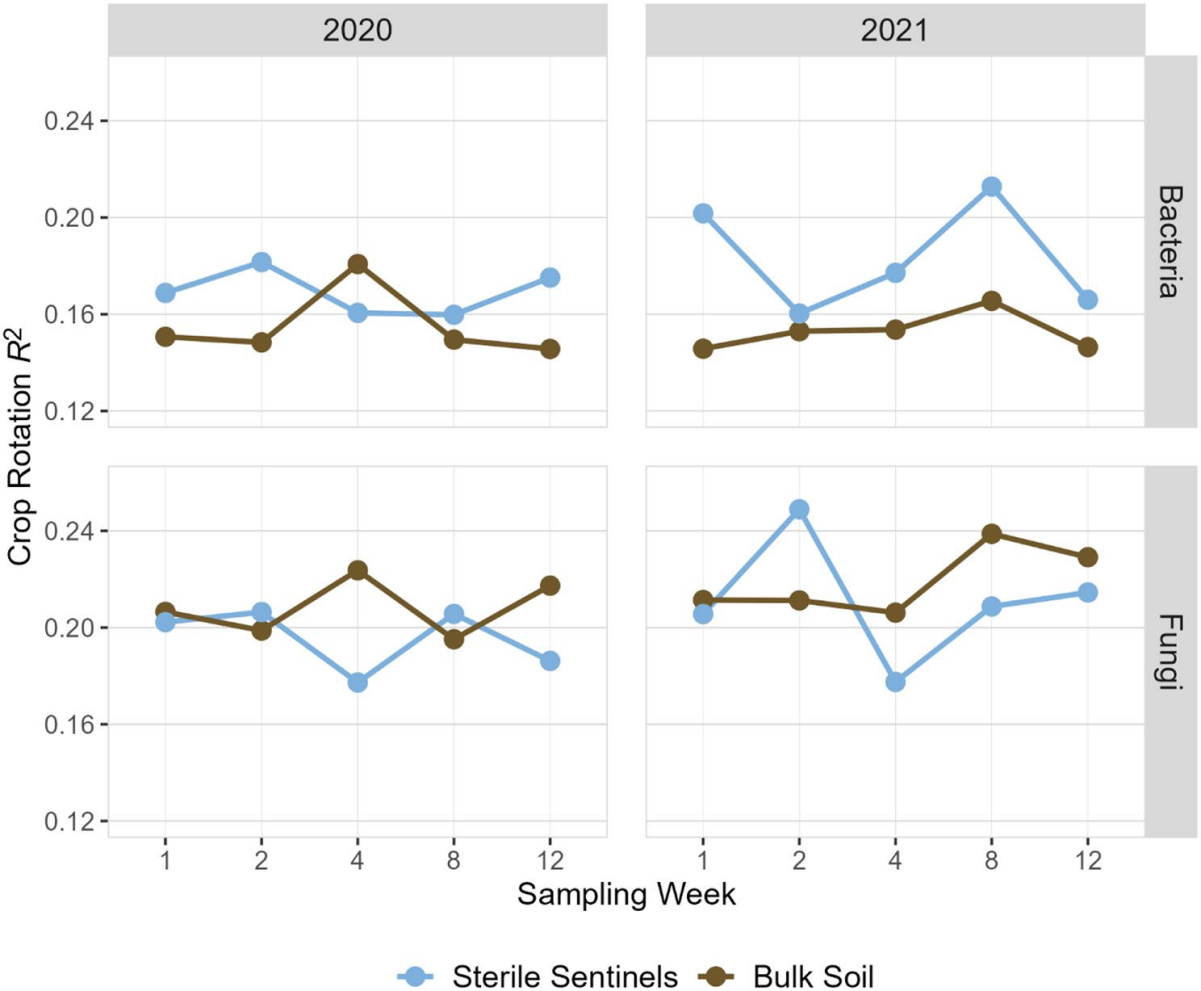
PermANOVA *R*^2^ from tests with crop rotation as the explanatory variable for community distance. PermANOVA tests were done for each sampling week (*n* = 4). A summary of each permANOVA test is in Supplementary Table 4

We hypothesized that sterile sentinels would capture a subset of the microbial community with higher statistical power to differentiate between crop rotations compared to bulk soil. Supporting this hypothesis, sterile sentinels had greater statistical power than bulk soil to distinguish bacterial community dissimilarity between crop rotations (Fig. 4; Table 1). For fungi, sentinels and bulk soil were not significantly different (Fig. 4; Table 1). Incubation time (sampling week) did not predict variance explained by crop rotation for sentinels (ANOVA, *p* > 0.01 for linear and polynomial time models).

**Table 1:** Paired t-test of bulk soil and sentinel PermANOVA *R*^2^ values for bacteria (*n* = 10) and fungi (*n* = 10). The paired t-test compared the 10 sentinel-bulk pairs from both years, seen in Fig. 4

Amplicon	Bulk Soil Mean *R*^2^	Sterile Sentinel Mean *R*^2^	t	Mean of Differences	*p*-value
Bacteria	0.154	0.176	3.316	0.022	0.009
Fungi	0.214	0.203	-1.332	-0.011	0.216

### Microbial trait and taxa differential abundance between sentinels and bulk soil

Supporting the expectation that taxa colonizing sentinels were more likely to be able to disperse and exploit new resources quickly, the differentially abundant microbial traits in sentinels were characteristic of copiotroph and ruderal life strategies. Taxa in sentinels also tended to be plant-associated (i.e., potential pathogens, symbiotic, potential endophytes). Sentinel bacteria had larger genomes and faster maximum growth rates than bulk soil bacteria for most sampling weeks in 2020 and 2021 (genome size: bulk soil mean = 5.18 × 10^6^, sentinels mean = 5.8 × 10^6^, t-test *p*-values < 0.01 for all sampling weeks; doubling time: bulk soil weighted mean = 9.9 h, sentinels weighted mean 1.9 h, Wilcoxon *p*-value < 0.01 for weeks 1–2. Supplementary Figs. 7–8). The total number of bacterial species assigned a genome size was 554 (45%) in 2020 and 412 (45%) in 2021 (Supplementary Table 5). The total number of bacterial species assigned a doubling time was 75 (6%) in 2020 and 45 (5%) in 2021 (Supplementary Table 5). Bacteria with potential gliding motility were more abundant in sentinels relative to bulk soil in sampling weeks 2–12, while potentially non-motile taxa were more abundant in bulk soil for all sampling weeks (absolute effect size > 1, Supplementary Fig. 9). The total number of bacterial species assigned a motility type was 439 (35%) in 2020 and 328 (36%) in 2021 (Supplementary Table 5). Bacterial taxa identified as plant pathogens were only present in sentinels (2020 = 14 taxa, 2021 = 12 taxa).

Fungal plant pathogens had higher abundance in sentinels than bulk soil at all sampling times in both years. Several other guilds were more abundant in sentinels relative to bulk soil in several sampling weeks, including soil saprotrophs (weeks 2–12), arbuscular mycorrhizae (weeks 2–8) and wood saprotrophs (8) in 2020. In 2021, mycoparasites were more abundant in sentinels relative to bulk soil for all sampling weeks. Pollen saprotrophs were more abundant in bulk soils than sentinels for weeks 2–12 (2021) and only week 12 in 2020 (for all differentially abundant guilds, absolute effect size > 1, Supplementary Fig. 10). The total number of fungal taxa assigned a guild was 1166 (54%) in 2020 and 948 (60%) in 2021 (Supplementary Table 5).

In total, 213 fungal and 569 bacterial taxa were differentially abundant between bulk soil and sentinels. Taxa highly abundant in bulk soil were not always the most abundant taxa in the sentinels, especially for bacteria (Supplementary Figs. 11–12). Bacterial genus *Massilia* was highly abundant in sentinels, as well as *Vitiosangium* and *Ramlibacter*. In contrast, genera *Gaiella*, *Chthoniobacter*, and *Vicinimibacter* were most abundant in bulk soils. Fungal taxa in the genera *Mortierella*, *Fusarium*, and *Bipolaris* were most abundant in sentinels, while *Phyllactinia*, *Podila*, and *Clonostachys* were most abundant in bulk soils.

### Microbial trait and taxa differential abundance between crop rotations

In contrast with hypotheses, microbial traits were not significantly different between crop rotations. Neither bacterial plant pathogens nor motility type showed significant differential abundance between crop rotations in sentinels or bulk soils (absolute effect size < 1). Bacterial genome size and maximum growth rate were not significantly different between CS and CSSwP rotations for either sample type (t-test, *p* > 0.05). Likewise, fungal guild differential abundance trends were weak with most effect sizes much smaller than or close to 1 for few sampling weeks in bulk soil and sentinels (Supplementary Fig. 13).

**Fig. 5:**
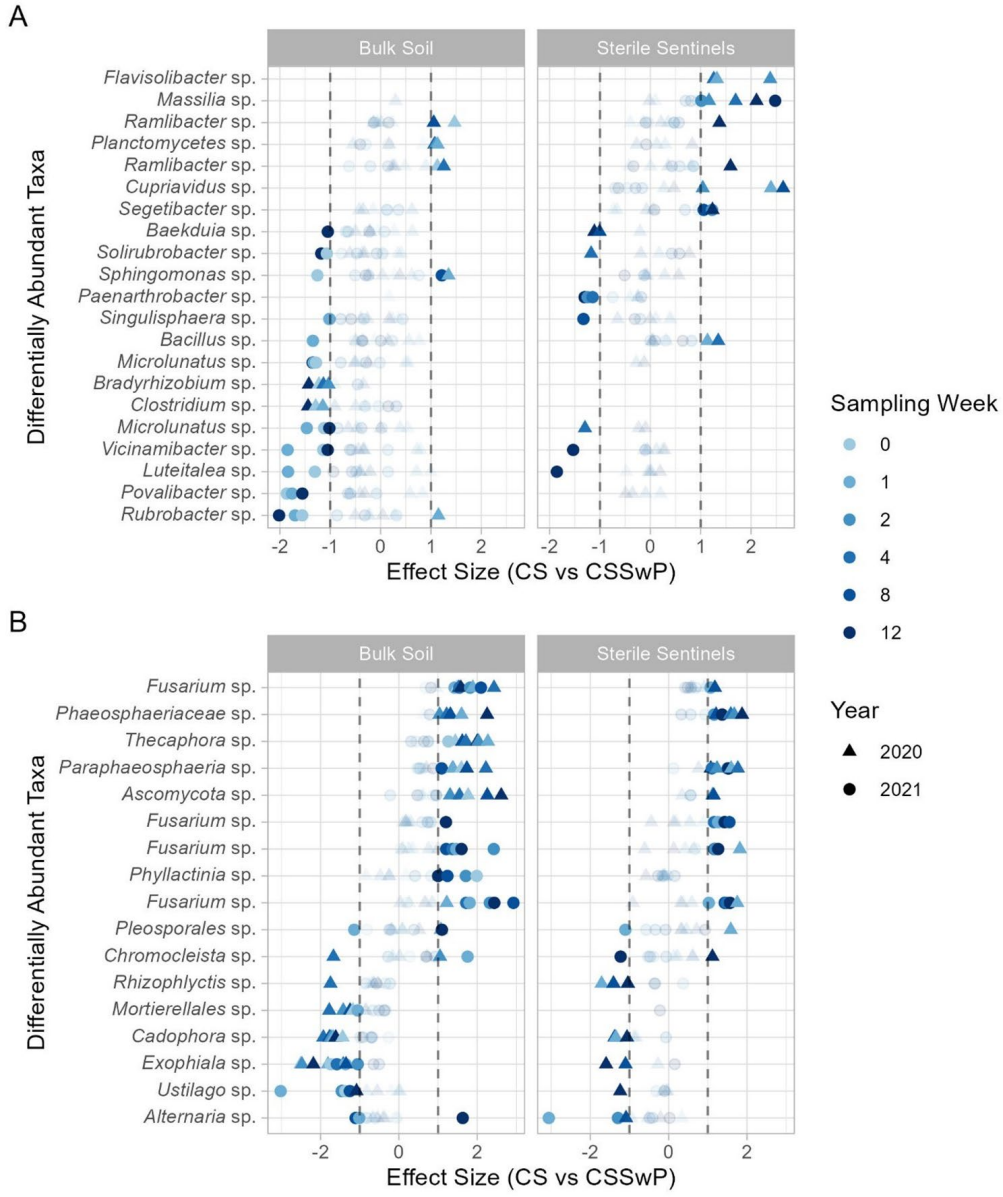
A subset of bacterial (**A**) and fungal (**B**) taxa differentially abundant between crop rotation. Each point represents the effect size of a single ALDEx2 t-test, i.e., differential abundance between CS and CSSwP for 2020 week 1 sentinel fungi (*n* = 4). Taxa with higher relative abundance in CS have negative effect sizes. The subset of taxa shown were chosen by ranking the taxa by total number of significant t-tests, then selecting most frequently differentially abundant taxa: bacteria had 21 taxa with 3 or more significant t-tests, fungi had 17 taxa with four or more significant t-tests. For the selected taxa, all t-test effect sizes are shown and t-tests with effect sizes less than 1 are shown with high transparency

Across all sample types, weeks, and both years, 194 unique fungal (81 genera) taxa and 327 unique bacterial taxa (238 genera) were differentially abundant between CS and CSSwP rotations. Emphasizing the validity of taxa collected in sentinels, the majority of sentinel and bulk taxa were differentially more abundant in the same rotation across sampling methods. For example, bacteria in the genera *Ramlibacter* (CSSwP) and *Microlunatus* (CS) and fungi in the genera *Paraphaeosphaeria* (CSSwP) and *Cadophora* (CS) (Fig. 5). However, a few taxa showed opposing patterns depending on sampling method, such as a *Bacillus* (bulk: more abundant in CS; sentinel: more abundant in CSSwP) (Fig. 5A). Each sampling methods also identified a smaller number of unique, differentially abundant taxa. For example, bacterial genera *Flavisolibacter* (CSSwP), *Cupriavidus* (CSSwP), and *Massilia* (CSSwP) were almost exclusively found in sentinels (Fig. 5A, total bacterial taxa differentially abundant in CSSwP: 2020 bulk 54, sentinel 96; 2021 bulk 21, sentinel 55). Conversely, *Rubrobacter* (CS), *Bradyrhizobium* (CS), and *Clostridium* (CS) were only differentially abundant in bulk soil (Fig. 5A, total bacterial taxa differentially abundant in CS: 2020 bulk 40, sentinel 36; 2021 bulk 78, sentinel 62). Sentinels captured more differentially abundant bacterial taxa than bulk soil that favored the CSSwP (*p* = 0.03, t-test), but not the CS rotation (*p* = 0.7, t-test). Still, comparing across both rotations, the total number of differentially abundant taxa that the sentinels captured was no different than the number that sampling bulk soil captured (paired t-test, t = -2.03, df = 9, *p* = 0.07, mean difference = -9.9).

In contrast, for fungi, bulk soils had more differentially abundant taxa between rotations than sentinels (paired t-test, t = -2.6, df = 9, *p* = 0.03, mean difference = 7.7). Bulk soils also captured more differentially abundant fungal taxa in CSSwP than sentinels (*p* = 0.003, t-test) but not in the CS rotation (*p* = 0.1, t-test). Overall, fungal genera frequently identified as differentially abundant in the CS rotation included the genera *Ustilago*, *Exophiala*, *Cadophora*, *Rhizophlyctis*. CSSwP rotations were characterized by *Thecaphora*, *Fusarium*, *Paraphaeosphaeria*, and *Phyllactinia* (Fig. 5B, total fungal taxa differentially abundant in CS: 2020 bulk 88 sentinel 37; 2021: bulk 38, sentinel 30 and in CSSwP: 2020 bulk 81, sentinel 45; 2021 bulk 59, sentinel 40).

## Discussion

In this study, we aimed to test the utility of sterile soil in-growth bags, sterile sentinels, for capturing an active, management-discriminating subset of the microbial communities in cropped soils. Comparing sentinels with concurrently sampled bulk soils, we demonstrated that sentinels distinguished crop rotations as well as or better than bulk soil, and that each sample type captured distinct microbial communities. The length of incubation time had no effect on predictive power of sentinels. However, the different incubation times did allow observation of taxa turnover and increased taxa richness in sentinels, including more shared species between the sentinels and bulk soil with longer sentinel incubation time. We did not detect significant differences in traits and functional groups between crop rotations from bulk soils or sentinels. However, taxonomic groups were differentially abundant between crop rotations. Overall, investigation of taxa identities and traits supported the hypothesis that the sentinels captured management-differentiating, active microbes. These microbes tended to be potential plant pathogens, root symbionts, endophytes, or rhizosphere taxa.

### Bacterial responses

Sentinels had greater power to detect crop rotation effects on bacterial communities. A potential mechanism behind this observation is that sentinels may have reduced the prevalence of dormant taxa relative to taxa that are actively growing near corn plants in soils with different crop rotation legacies. Dormant taxa can comprise up to 60% of soil DNA [7] while dead organisms can use valuable read space within high throughput sequencing methods [9]. Sentinel bacterial richness and diversity was higher than or equal to bulk soil, even as total DNA was substantially lower than in bulk soil, suggesting that the sentinels served as a “hot spot” for bacterial growth for bacteria already more active in the bulk soil due to the proximity of living plant roots [28]; the sentinels were place in the crop row. The higher abundance of plant-associated bacteria, such as rhizosphere taxa *Massilia* (Ofek et al., 2012) and plant pathogens, in sentinels also support this interpretation. Therefore, while crop rotation did influence bulk soil communities, explaining up to 15% of differences in community composition (Fig. 4), the additional 2-2.5% of bacterial community variation explained by sentinels could be due to detection of taxa that are likely to be interacting with crops.

Bacteria colonizing sentinels offered distinct and complementary views of community response to crop rotation compared with bulk soil. Bacteria in sentinels had traits that correlated with copiotrophic or ruderal taxa such as high potential maximum growth rate, motility, and large potential genome size [20, 24, 43], Wood et al., 2023). Motility, maximum growth rate, and genome size are also critical traits for rhizosphere bacterial taxa [34]. Both the proximity of sentinels to plant roots and the fact that early colonizing bacteria of barren substrates and rhizosphere bacteria may share similar traits may be one explanation for why the sentinels captured actively growing, plant-associative bacteria. Sentinel bacteria were also more likely than bulk soil bacteria to be plant pathogens. These observations suggest that sentinel communities can be useful for evaluating crop management. Sentinels may facilitate monitoring for disease prevention or allow tracking soil responses to management designed to reduce pathogen load, for example crop rotation [4]. We did not observe hypothesized differences in bacterial traits between rotations. Several potential explanations could account for no differences: lack of power due to the number of taxa with trait data, the traits we investigated are not relevant to the rotations, or greater sequencing depth was needed to detect differences.

### Fungal responses

Fungal communities colonizing sentinels also offered complementary views of overall community responses. As with bacteria, sentinels were selective for plant pathogens and plant mutualists compared to bulk soil. For example, arbuscular mycorrhizal fungi and the plant-associated genera *Bipolaris* (pathogen), *Mortierella* (endophyte-soil saprotroph), and *Fusarium* (pathogen or beneficial) were more abundant in sentinels than bulk soils. Sentinel fungi were neither more nor less sensitive to management than bulk fungi, in contrast with bacteria. This difference could be due to life history traits including growth form, slower growth, and greater investment in persistent structures relative to bacteria, or a lower competitive advantage with bacteria in the relatively homogenous and resource-rich autoclaved soil in the sentinels, or fewer truly active fungi in the soil [53]. Fungal richness and diversity were lower in sentinel than bulk soils, suggesting that only a few fungi sought or were able to colonize sterile sentinels during the incubation period. A previous study of arbuscular mycorrhizal fungi in sand in-growth bags vs. bulk soils also detected fewer fungi in in-growth bags, but these fungi still captured treatment effects [61], as we found in our study.

Overall, we did not find differences in functional guilds between crop rotations. Both rotations hosted similar functional guilds, yet these guild were comprised of different species, and we did find consistent functional patterns when examining differentially abundant taxa. For example, differentially abundant CS fungal taxa tended to be stress tolerating endophytes (*Exophiala, Mortierella*) [67], arbuscular mycorrhizal fungi (*Corymbiglomus*), and plant pathogens of corn and soybean (*Ustilago*, *Cadophora*). In comparison, the CSSwP system contained a different set of plant pathogens (*Phyllactinia*, *Fusarium*, *Thecaphora*) and plant endophytic fungi (*Paraphaeosphaeria*, *Septoglomus*, *Fusarium*). We also observed expected patterns that underlie benefits of diverse crop rotations. For example, *Cadophora gregata*, the cause of brown stem rot of soybean [25], was more abundant in CS than CSSwP rotations. This pathogen does not decline in abundance over the corn growing season in CS but is barely present in CSSwP, supporting the role of more diverse rotations in reducing pathogen pressure and the importance of previous crop and crop legacies in determining soil microbial communities [4, 5, 46, 57].

### Bulk soil microbial community differences between crop rotations

Across two growing seasons and multiple sampling times per season, analysis of bulk soils established that CS and CSSwP crop rotations featured distinct bacterial and fungal communities, but we found no consistent differences in diversity or traits between rotations. A meta-analyses of crop rotation effects on soil microbial diversity reported a 3% increase in microbial diversity with increased rotational diversity [62]. However, only a few studies report bulk soil microbial community composition and diversity response to crop rotational diversity that were conducted in agricultural systems with more than two crops in long-term, fully replicated field trials. These studies report that crop rotation diversity significantly alters bacterial community composition [46, 52, 57], with which our study agrees. Microbial diversity has a more complex relationship with crop rotational diversity; studies report that bulk soil microbial diversity increases [68], decreases [46], or does not change [52, 57] with increased crop rotational diversity. Given our results and those from the literature, crop rotational diversity shapes soil microbial community composition with context, or scale dependent effects on microbial diversity, although there is a need for more data on fungi in long-term crop rotations with more than two crops.

### Use of the MinION sequencer for metabarcoding of soil microbial communities

We used a third generation, long read sequencer from Oxford Nanopore in this study. Several advantages and disadvantages of this sequencing method are apparent that several papers have already discussed, including quick sample-to-data turnaround, longer reads, and higher error rates [6, 16, 26, 35, 60]. In our experience, preparing the libraries and sequencing with the MinION was easy and quite fast compared to the longer turnaround time from sequencing centers. We agree that it is an excellent tool for rapid response monitoring of targeted organisms, particularly because of the read length. The kilobase and longer reads allow sequencing the full 16 S rRNA gene to achieve species-level resolution for bacteria [40]. Longer reads allow the variable fungal ITS marker to be flanked by more the more conservative 18 S and 28 S, which could aide phylogenetic placement and species level distinction for some taxa [37].

These benefits come with tradeoffs. The MinION produces relatively few reads, which is problematic for adequately sampling the hyper diverse communities found in soil. However, less diverse communities or lower DNA loads as observed for the sterile sentinels could be well served by a MinION, especially for monitoring a small set of organisms. The nanopore error rates and bias are a point of concern for some metabarcoding applications [35]. Our use of a positive control allowed us to mitigate these concerns in part [55], as we were used a mock community as a benchmark for bioinformatics choices. As Oxford Nanopore accuracy improves, we expect that long read sequencing with rapid sample-to-data turnaround for metabarcoding will become widely used.

### Applications of sentinels to monitoring and managing soil microbial communities

Our results suggest that combining sterile sentinels with long-read sequencing could aid in reproducibly monitoring and actively managing microbial communities. A monitoring-active management scenario requires a method that (a) returns data rapidly; (b) selects for actively growing taxa; and (c) is easily and reproducibly executed across contexts, given any level of user experience.

Regarding rapid turnaround, farmers require relevant feedback about the effects of their management choices on microbes – particularly regarding pathogen pressure – within days of sampling [19]. A simple, fast assay like sterile sentinels, paired with rapid sequencing, such as via MinION, can provide this turnaround. The benefits of MinION sequencing must be considered along with the challenges and drawbacks noted above.

Sentinels seem to select for actively growing, plant-associated taxa. Sentinels have multiple advantages and limitations compared to methods that were designed to provide similar data about soil microbial communities, such as standard bulk soil, soil RNA or plant rhizospheres. Although RNA is used to profile the active community, RNA levels may better represent past, current, and future potential activity rather than current activity [71]. Moreover, the instability of RNA can make accurate sampling technically and logistically difficult. In contrast, sentinels are easy to use and do capture actively growing microbes, although it is a limited subset of the entire community. As an alternative to RNA analyses, researchers may exclude relic DNA, the extracellular DNA from dead microbial cells, to better estimate the living soil community. Relic DNA has the potential to affect estimates of microbial diversity and dynamics and may be excluded through chemical treatments before DNA extraction Lennon et al. [70], Carini et al [9]. In contrast, sentinels were designed to subsample the microbial community before relic DNA accumulates.

Finally, we expect sentinel results to be more transferrable because the process of manufacturing, placing, and removing sentinels reduces sources of technical variation including of soil type, sampling depth, and sampling volume. These are major sources of variation in soil microbial ecology studies [10, 66]. For example, using a standard soil could reduce the technical variability of extracting microbial DNA from different soil types [70]. Reducing these sources of error and bias will allow for specific and robust comparisons of microbial communities across sampling and ecological contexts.

This sterile sentinel method can be used to distinguish the actively growing microbial communities among experimental treatments when a highly standardized, cost-effective method is needed. The design of the sentinels could be modified for exclusion or inclusion of different groups. We excluded roots and included fungi and bacteria by using 31 μm nylon mesh [27], but mesh size could be altered to address different fractions of the microbial community or include plant roots, to suit the research question. For example, although focused on leaf litter, Albright and Martiny [3] used different mesh sizes (18 μm and 0.22 μm) to manipulate bacterial dispersal rates, as well as different substrates in the mesh bags, to disentangle the roles of dispersal, growth rate, and succession in bacterial community diversity and composition. Alternatively, using a larger mesh size to include plant roots may allow direct collection of rhizosphere communities.

Further investigation of sentinels across incubation timing and duration, and across soil types and depths could test the utility of the sentinels beyond distinguishing between experimental treatments. Questions could include: how meaningful is the sampled community for indicating ecological processes, such as nutrient cycling? How do sentinel results of potential soil microbial activity compare with rhizosphere communities? Do sentinel microbial communities predict plant health, or can sentinels be used to monitor plant health? And to what extent are these results consistent across soil types and stochastic weather patterns?

## Conclusions

Soil bacteria and fungi are critical to soil processes and plant growth. In agricultural systems, this translates to significant influence on agronomic and environmental outcomes. Still, assessing the active component of these communities – the taxa responding to management, interacting with plants, and cycling nutrients – remains challenging. The sterile sentinel method attempts to improve assessment by incubating uncolonized and standard soil within a root exclusion bag in the field. The method provided greater resolution in identifying management effects on bacterial community structure and allowed inference of management effects on bacteria and fungi. Sentinel results therefore provided complementary information about soil microbial communities to that of bulk soil. Further testing across sites and deployment schemes will enable assessment of the transferability of these results. The method also allowed easy and standardized sample collection without specialized equipment. When combined with rapid-turnaround sequencing technologies, we anticipate this method could facilitate routine and large-scale assessment of critical soil microbes, ultimately leading to better prediction and management of their role in agricultural systems.

### Electronic supplementary material

Below is the link to the electronic supplementary material.

Supplementary Material 1

## Data Availability

Sequencing data used in this paper is available in the NCBI SRA under BioProject number PRJNA1020132. Bioinformatics scripts, R analysis code, and data tables are available at: https://github.com/serlandson/sterile_sentinels.
