# Supplementary material for: Sterile sentinels and MinION sequencing capture active soil microbial communities that differentiate crop rotations
Source: Environ Microbiome. 2024 May 7;19:30. doi: 10.1186/s40793-024-00571-8 (PMC11077770; doi:10.1186/s40793-024-00571-8)
Supplement: Supplementary file 1 — Supplementary Material 1 [file 40793_2024_571_MOESM1_ESM.pdf]

Supplementary Table 1. Read and taxa counts for each sequencing library. Filtered Emu and minimap2 taxa tables were obtained by removing reads or taxa that were unclassified, only present in negative controls, represented by less than 3 reads, or matched to chloroplasts or mitochondria. Only taxa that matched to Kingdom Fungi were retained in Eukaryotes dataset. Controls included DNA extraction negative control, PCR1 negative control, PCR2 negative control, ZymoBIOMICS Microbial Community DNA Standard (#D6305) positive control, and autoclaved soil control.

	**Bacteria**		**Eukaryotes**	
**Read Count**	**2020**	**2021**	**2020**	**2021**
Total Initial	3575783	1252751	3144515	4761322
Quality Filtered	1850995	963088	2410496	1699959
Emu Assigned	1707735	884952	558997	209610
Emu Unclassified	270	165	48601	3559
Minimap2 Classified	792395	386796	693788	338150
Minimap2 Unclassified	1058600	576292	1716708	1361809
**Taxa**				
Emu raw output	1475	970	266	352
Emu filtered table	1240	920	223	306
Minimap2 raw output	30389	23262	7937	6079
Minimap2 filtered table	14564	14836	2173	1580

Supplementary Table 2. Summary of mean richness and diversity in the two sample types for each amplicon and year. T-tests compared the sentinels to bulk soils within each dataset (n = 20, excluded week 0 bulk soil from t-tests).

	**Richness (Hill *q* = 0)**					**Diversity (Hill *q* = 1)**				
	**Sentinels**		**Bulk Soil**			**Sentinel**		**Bulk Soil**		
**Dataset**	**Mean**	**SD**	**Mean**	**SD**		**Mean**	**SD**	**Mean**	**SD**	
**Fungi 2020**	184	30	346	19	**	44	12	120	20	**
**Fungi 2021**	149	19	212	25	**	31	9	69	23	**
**Bacteria 2020**	299	64	278	77		146	33	100	23	**
**Bacteria 2021**	164	41	138	44	*	74	34	59	17	*

**Indicates sentinel vs. bulk soil t-test significant at $p < 0.005$, *at $p < 0.05$

Supplementary Table 3. Redundancy Analysis (RDA) results summary for ordinations shown in Figure 3 and Supplementary Figure 5. The RDA constraining variables were sampling week and rotation. RDA tests used the adonis2 function in vegan, with a PermANOVA test of marginal and axis significance. Axis results are shown below.

Bacteria 2020 – Constraints Explained 57% of Total Variance

	df	Variance	*F*	p-value	Prop. Variance
RDA1	1	953.51	106.3623	0.001	0.54
RDA2	1	45.65	5.092	0.001	0.026
RDA3	1	13.39	1.4933	0.056	0.008
Residual	84	753.04			

Bacteria 2021 - Constraints Explained 57% of Total Variance

	df	Variance	*F*	p-value	Prop. Variance
RDA1	1	663.11	99.7533	0.001	0.525
RDA2	1	40.53	6.0965	0.001	0.032
RDA3	1	13.55	2.0382	0.001	0.011
Residual	82	545.09			

Fungi 2020 - Constraints Explained 38% of Total Variance

	df	Variance	*F*	p-value	Prop. Variance
RDA1	1	177.05	42.1247	0.001	0.313
RDA2	1	23.06	5.4874	0.001	0.041
RDA3	1	12.82	3.0513	0.001	0.023
Residual	84	353.05			

Fungi 2021 - Constraints Explained 45% of Total Variance

	df	Variance	*F*	p-value	Prop. Variance
RDA1	1	133.793	59.7327	0.001	0.399
RDA2	1	10.942	4.8853	0.001	0.033
RDA3	1	7.24	3.2323	0.001	0.022
Residual	82	183.669			

Supplementary Table 4. PermANOVA summary for each test used to make Figure 4, which tested the "variance explained by sentinel vs bulk soil" hypothesis. For each week, rotation was used as the predictor of community distance. Tests with significant p-values ($p < 0.05$) are in bold. The R^2 values were used to calculate means in the t-test summarized in Table 1 in the main text. Two samples were discarded due to sample mislabeling in 2021: one replicate from CS bulk soil week 0, and one replicate from CSSwP sentinel week 1.

Bacteria 2020

**Bulk Soil**					**Sentinels**				
**Week**	**Total df**	R^2	F	**p-value**	**Week**	**Total df**	R^2	F	**p-value**
0	7	0.15	1.1	0.208					
1	7	0.15	1.06	0.256	1	7	0.17	1.22	0.09
2	7	0.15	1.05	0.336	**2**	**7**	**0.18**	**1.33**	**0.033**
**4**	**7**	**0.18**	**1.32**	**0.026**	**4**	**7**	**0.16**	**1.15**	**0.025**
8	7	0.15	1.05	0.302	8	7	0.16	1.14	0.151
12	7	0.15	1.02	0.348	**12**	**7**	**0.18**	**1.27**	**0.031**

Bacteria 2021

**Bulk Soil**					**Sentinels**				
**Week**	**Total df**	R^2	F	**p-value**	**Week**	**Total df**	R^2	F	**p-value**
0	6	0.2	1.23	0.051					
1	7	0.15	1.02	0.381	1	6	0.2	1.26	0.087
2	7	0.15	1.08	0.261	2	7	0.16	1.15	0.215
4	7	0.15	1.09	0.231	4	7	0.18	1.29	0.07
8	7	0.17	1.19	0.141	**8**	**7**	**0.21**	**1.62**	**0.039**
12	7	0.15	1.03	0.325	12	7	0.17	1.19	0.118

Fungi 2020

**Bulk Soil**					**Sentinels**				
**Week**	**Total df**	R^2	F	**p-value**	**Week**	**Total df**	R^2	F	**p-value**
**0**	**7**	**0.2015**	**1.514**	**0.022**					
**1**	**7**	**0.2065**	**1.561**	**0.026**	**1**	**7**	**0.202**	**1.521**	**0.025**
**2**	**7**	**0.1988**	**1.489**	**0.037**	**2**	**7**	**0.206**	**1.561**	**0.031**
**4**	**7**	**0.2237**	**1.729**	**0.018**	**4**	**7**	**0.177**	**1.293**	**0.031**
**8**	**7**	**0.1952**	**1.455**	**0.027**	**8**	**7**	**0.206**	**1.554**	**0.031**
**12**	**7**	**0.2173**	**1.666**	**0.032**	12	7	0.186	1.373	0.069

Fungi 2021

**Bulk Soil**					**Sentinels**				
**Week**	**Total df**	R^2	F	**p-value**	**Week**	**Total df**	R^2	F	**p-value**
**0**	**6**	**0.2392**	**1.572**	**0.045**					
**1**	**7**	**0.2114**	**1.609**	**0.023**	1	6	0.206	1.294	0.081
**2**	**7**	**0.2112**	**1.607**	**0.025**	**2**	**7**	**0.249**	**1.989**	**0.028**
**4**	**7**	**0.2062**	**1.558**	**0.022**	4	7	0.178	1.295	0.052
**8**	**7**	**0.2388**	**1.882**	**0.044**	**8**	**7**	**0.209**	**1.583**	**0.025**
**12**	**7**	**0.2291**	**1.783**	**0.033**	**12**	**7**	**0.215**	**1.639**	**0.035**

Supplementary Table 5. Numbers of bacterial and fungal taxa (species) assigned to each trait by sample type and week. Total taxa with genome size data for each year were 544 in 2020 and 412 in 2021. Total taxa with doubling time data were 75 in 2020 and 45 in 2021. Total taxa with a motility trait assigned were 439 in 2020 and 328 in 2021. Total taxa with a fungal guild assignment were 1166 in 2020 and 948 in 2021.

**Bacteria**	**2020**		**2021**	
	**Bulk Soil**	**Sterile Sentinel**	**Bulk Soil**	**Sterile Sentinel**
**Sampling Week**	**Genome Size**			
**1**	236	199	131	138
**2**	229	228	123	179
**4**	247	233	140	147
**8**	187	253	102	147
**12**	233	214	118	186
**All Weeks Mean**	226	225	123	159
**Standard Deviation**	21	18	13	19
	**Doubling Time**			
**1**	48	14	23	8
**2**	43	21	23	10
**4**	49	25	21	11
**8**	38	35	20	13
**12**	46	28	19	16
**All Weeks Mean**	45	25	21	12
**Standard Deviation**	4	7	2	3
	**Motility**			
**1**	169	174	100	109
**2**	169	196	91	147
**4**	181	196	103	126
**8**	140	198	75	123
**12**	175	171	89	151
**All Weeks Mean**	167	187	92	131
**Standard Deviation**	14	12	10	16

**Fungi**				
**Sampling Week**	**Guild**			
**1**	694	402	513	355
**2**	668	434	480	377
**4**	696	499	558	431
**8**	669	499	497	382
**12**	686	463	465	445
**All Weeks Mean**	683	459	503	398
**Standard Deviation**	12	38	32	34

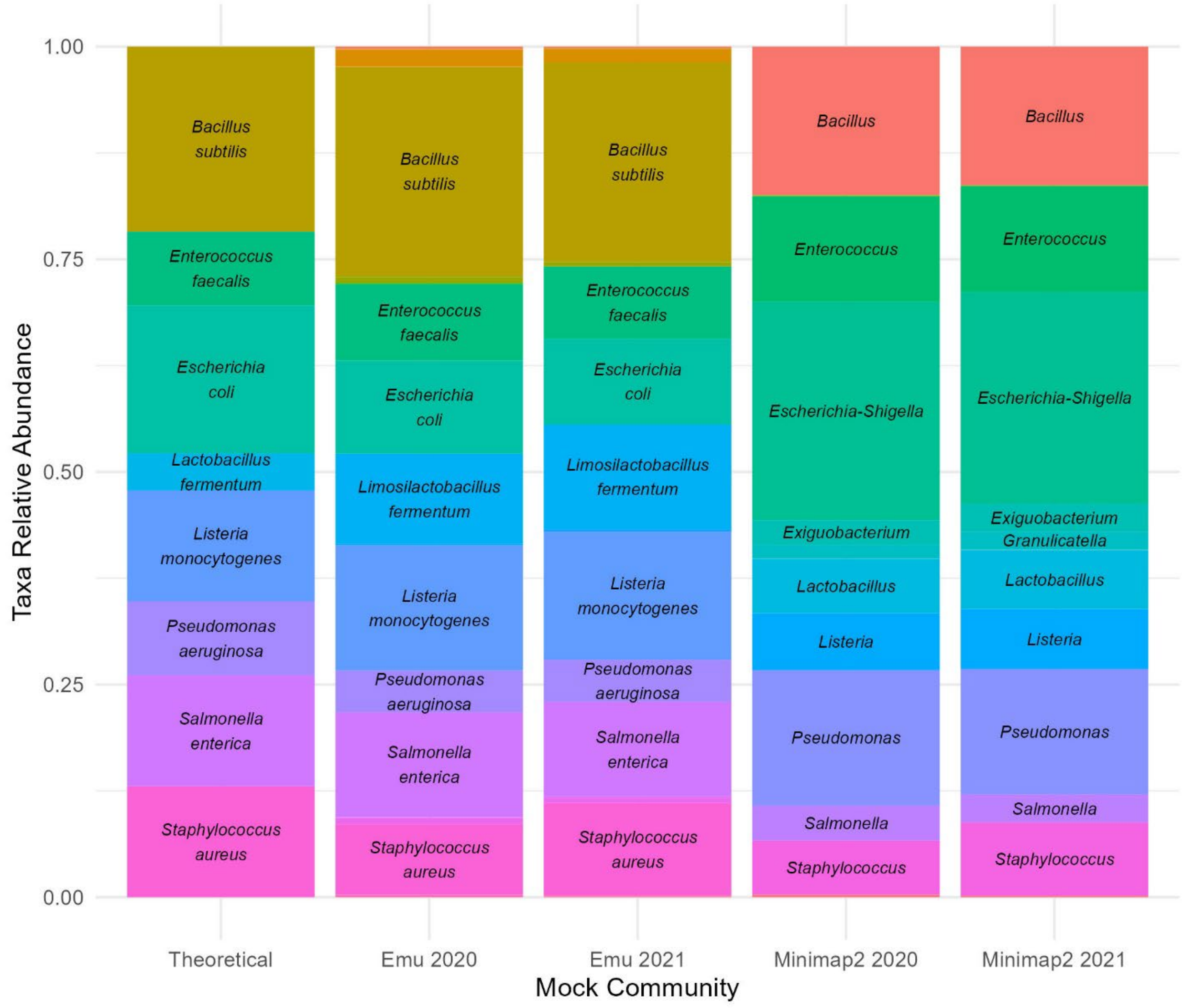

Supplementary Figure 1. Bacteria mock community taxa relative abundance for 2020 and 2021 using Emu or minimap2 to map MinION reads to 16S databases compared with the theoretical mock community relative abundance. Minimap2 reads are shown at genus level because the numbers of "species" were large and difficult to see on a plot. The reference databases used were the default Emu database for Emu and Silva version 138 for minimap2.

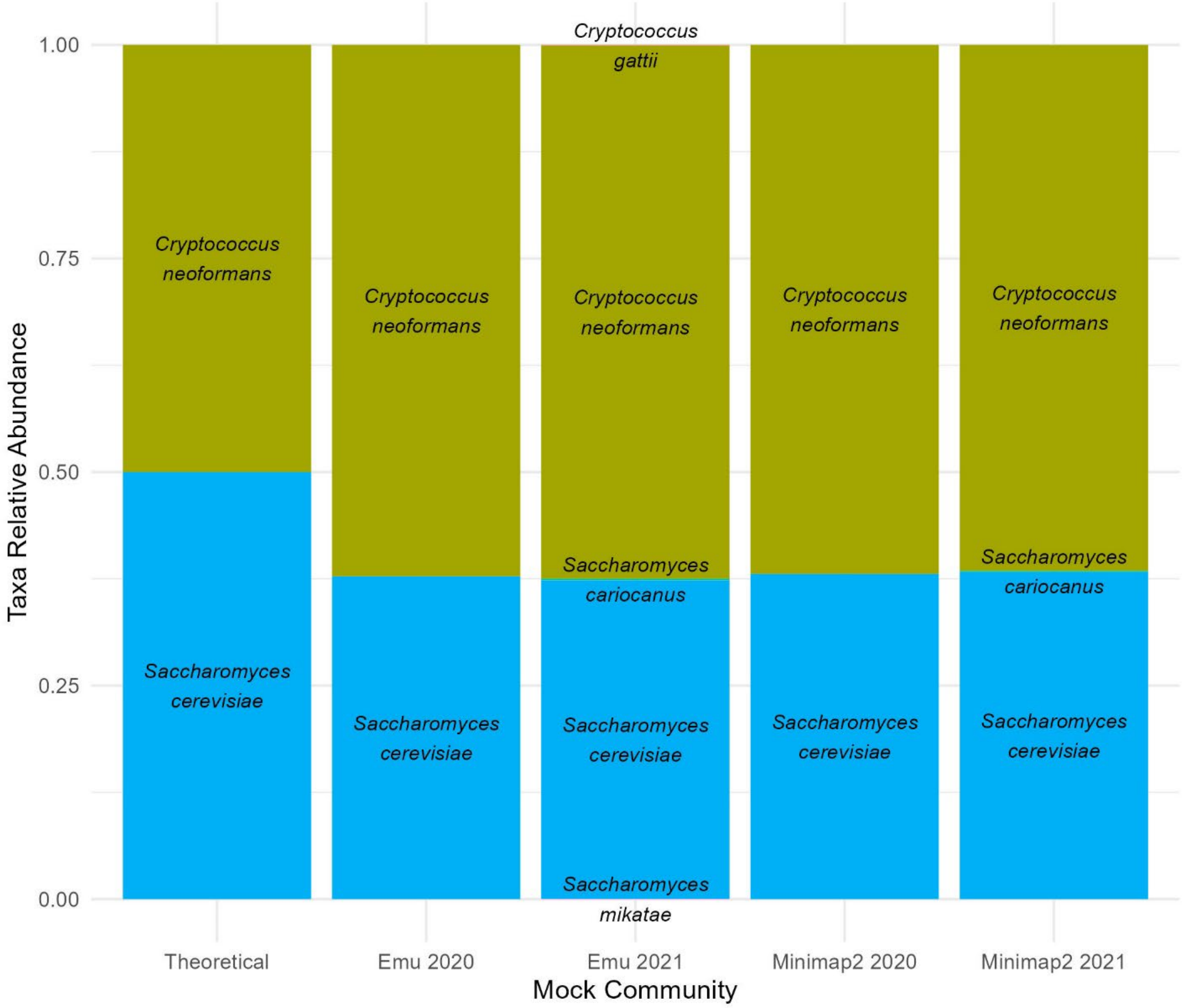

Supplementary Figure 2. Theoretical fungal mock community taxa relative abundance compared to the two bioinformatics approaches, Emu and minimap2, for 2020 and 2021 libraries. The mock communities were similar between Emu and minimap2, but minimap2 retained more reads. The same UNITE all eukaryotes 29.11.2022 database was used for both programs. The UNITE database was formatted for Emu using Emu's python script in the supplementary scripts.

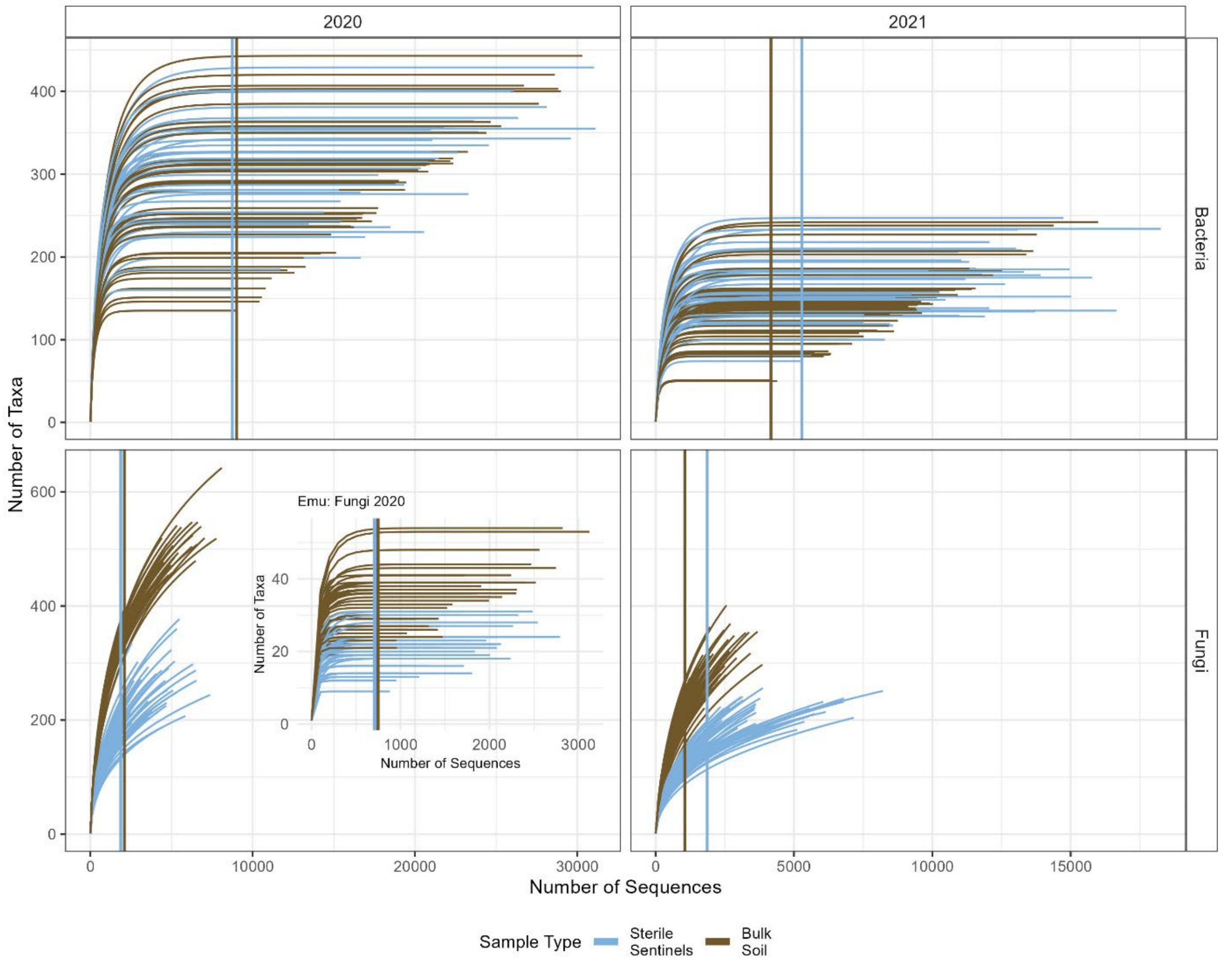

Supplementary Figure 3. Rarefaction curves for each dataset, bacteria reads were grouped with Emu and fungal reads were grouped by mapping reads to a reference database with minimap2. Each curve represents one sample, and the vertical lines represent the rarefied read count (the sample with the lowest read count). Bacteria appear sufficiently sampled, while fungi appear to require deeper sequencing depth. However, the shape of the curves is affected by read grouping method, as seen in the inset plot of Fungi 2020 sequencing reads grouped with Emu. Emu tends to reduce the numbers of rare taxa through error correction, while it is possible that grouping reads with minimap2 overestimates rare taxa. Rarefaction curves are likely not appropriate for Emu-processes reads.

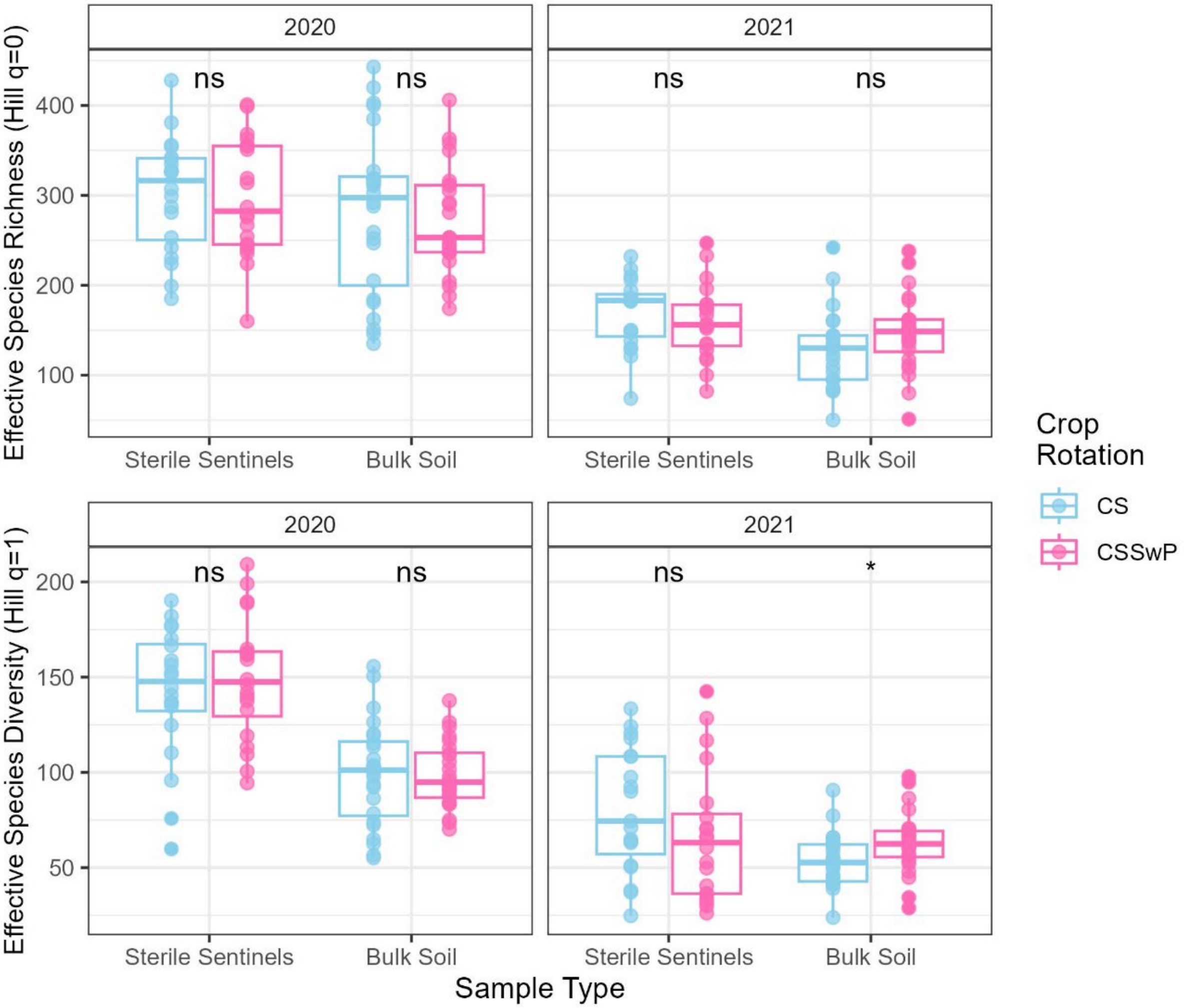

Supplementary Figure 4. Bacterial richness (A) and diversity (B) summarized by sample type and rotation. No significant differences in richness and diversity were found between CS and CSSwP crop rotations treatments for sterile sentinels. The CSSwP rotation had marginally higher diversity than CS in 2021 bulk soil ($p = 0.04$). Richness was lower overall in 2021 than 2020. Bulk soil richness was slightly lower than sentinels in 2021 ($p = 0.05$).

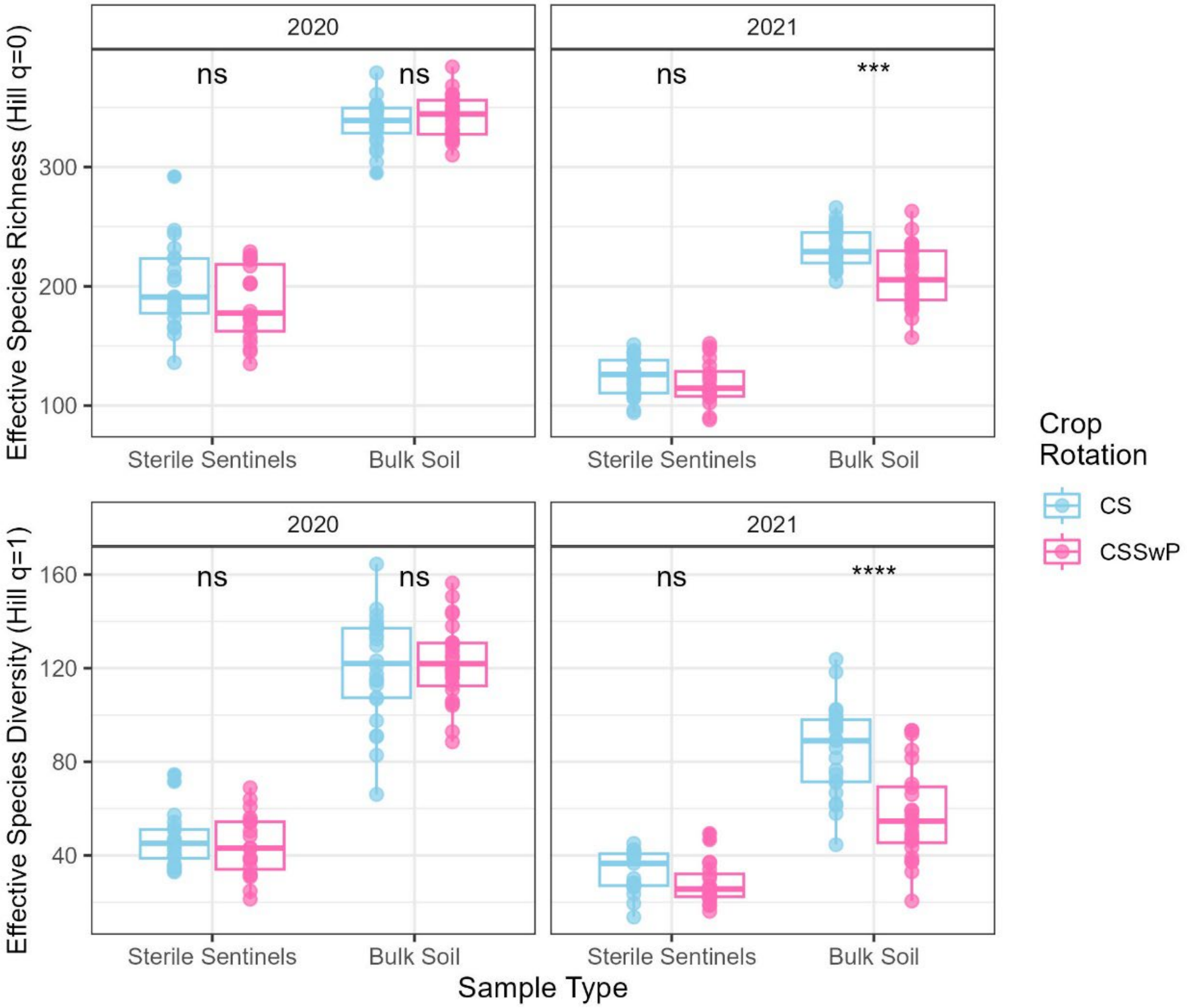

Supplementary Figure 5. Fungal richness (A) and diversity (B) over a 12-week incubation period for sterile sentinels and bulk soil in 2020 and 2021. Fungal richness and diversity were lower in sentinels than bulk soil ($p < 0.005$). T-tests of differences in richness and diversity between rotation including all time points were only significant for bulk soil in 2021 with higher richness and diversity in CS rotations ($p = 0.005$). The amount of DNA extracted from these 2021 samples was also lower in CSSwP rotations.

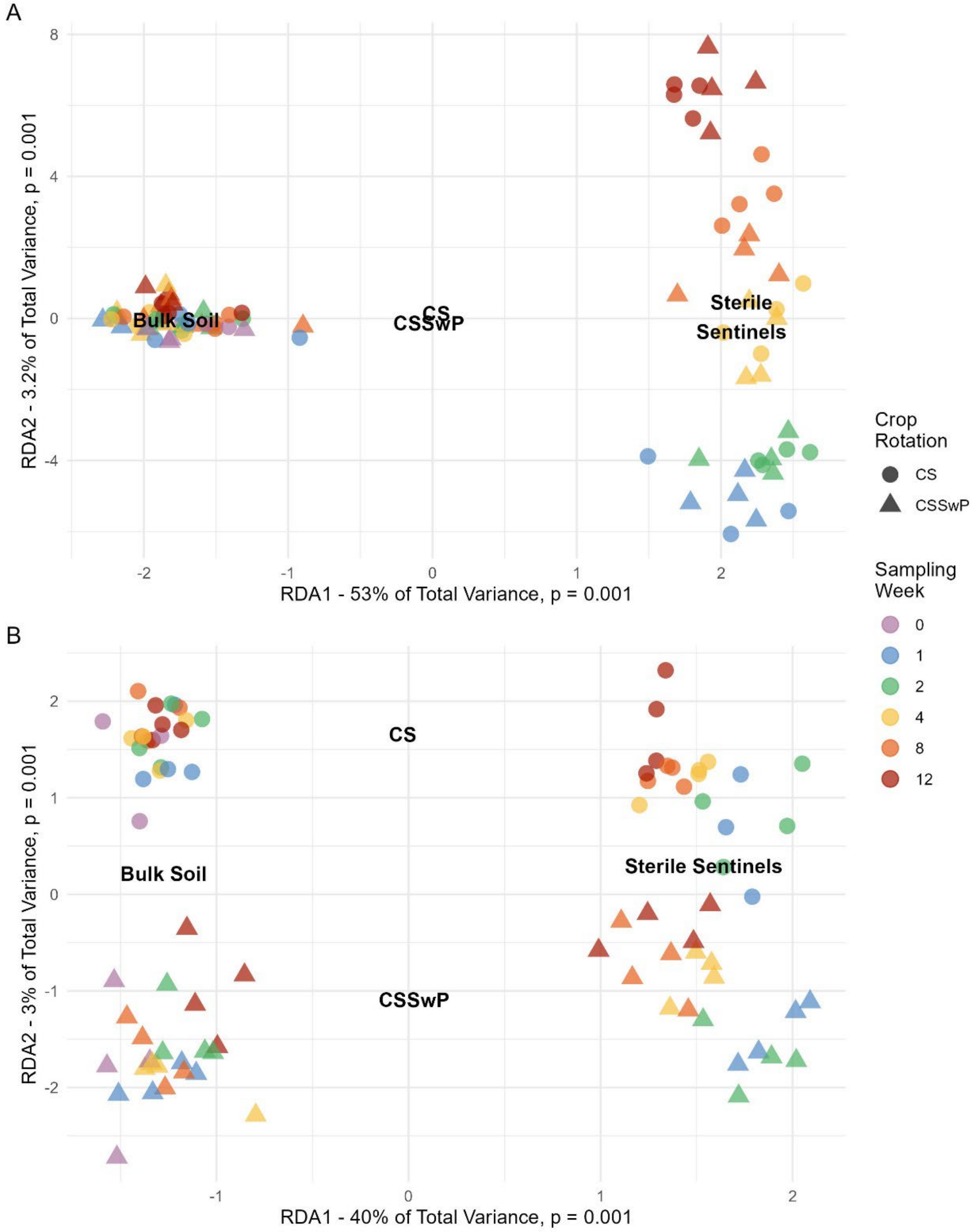

Supplementary Figure 6. Bacterial RDA ordination for 2021 (A) and fungal RDA ordination for 2021 (B) with sample type, sampling week and crop rotation as predictors. Colored points represent individual samples. The amount of constrained variance explained by each RDA axis is indicated along with significance. Text indicates centroids for categorical predictors sample type and crop rotation. The RDA ordinations reveal differences in predictor hierarchy. Variance in bacterial community composition is best explained by sample type, followed by sampling week, and finally crop rotation, while fungal composition is best described by sample type and crop rotation, then sampling week.

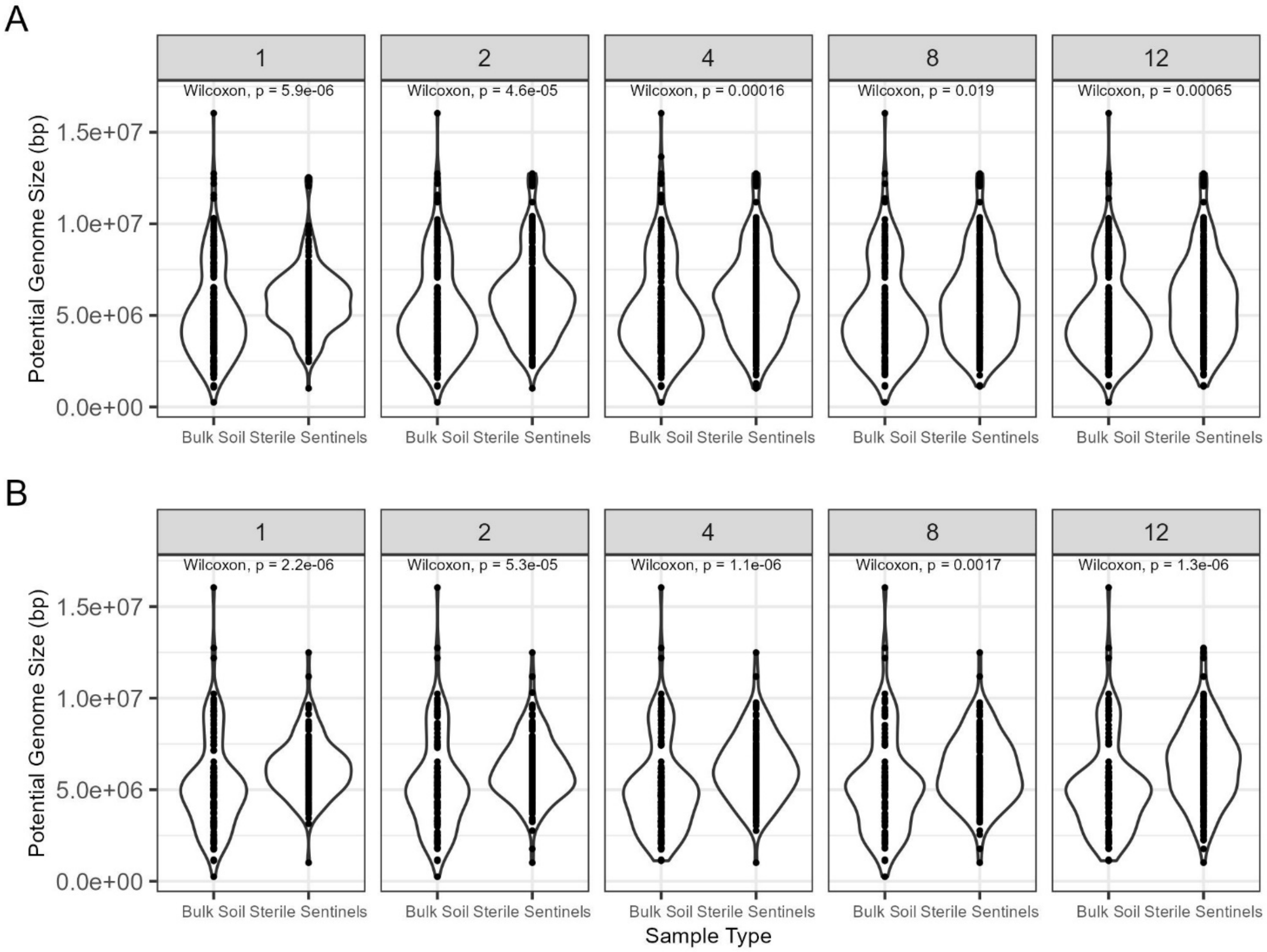

Supplementary Figure 7. Bacterial predicted genome size for each sample type within sampling week (1, 2, 4, 8, 12). Bacteria predicted genome size was significantly larger in sterile sentinels than bulk soil in 2020 (A) and 2021 (B). T-tests for each week in both years were significant at $p < 0.01$. Predicted genome size data was assigned to taxa according to the data compiled by Madin et al. 2020, https://doi.org/10.1038/s41597-020-0497-4. Sources included Genbank, GOLD and others.

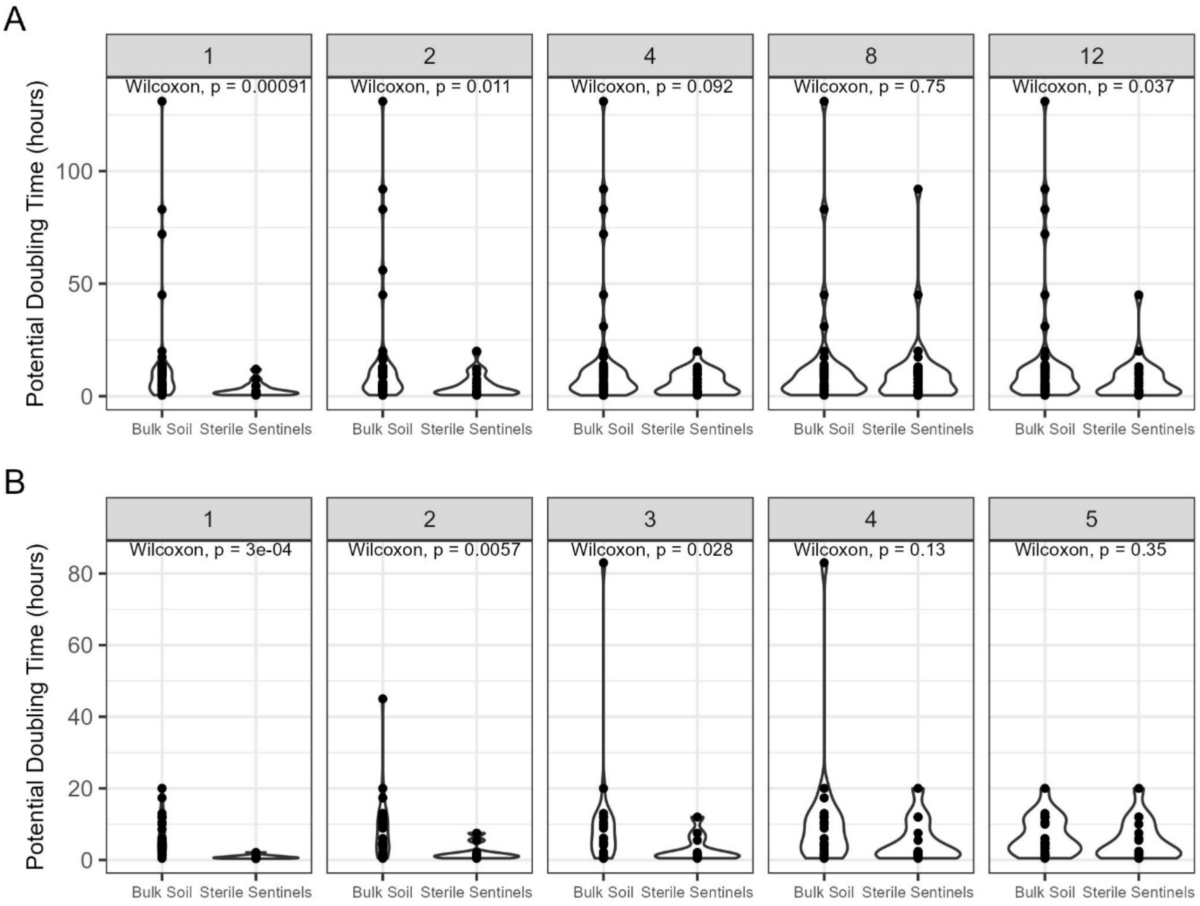

Supplementary Figure 8. Bacteria potential doubling time for each sample type within sampling week (1, 2, 4, 8, 12). Bacteria with longer potential doubling times are less represented in sterile sentinels compared to bulk soil in 2020 (A) and 2021 (B). Over the course of incubation, bacteria with longer potential doubling times appear in sentinels. T-tests were significant for weeks 1 and 2 in both years at $p < 0.01$, and significant including all time points ($p < 0.05$). Growth rate data was assigned to taxa according to the data compiled by Madin et al. 2020, https://doi.org/10.1038/s41597-020-0497-4, which represents growth rates in laboratory conditions.

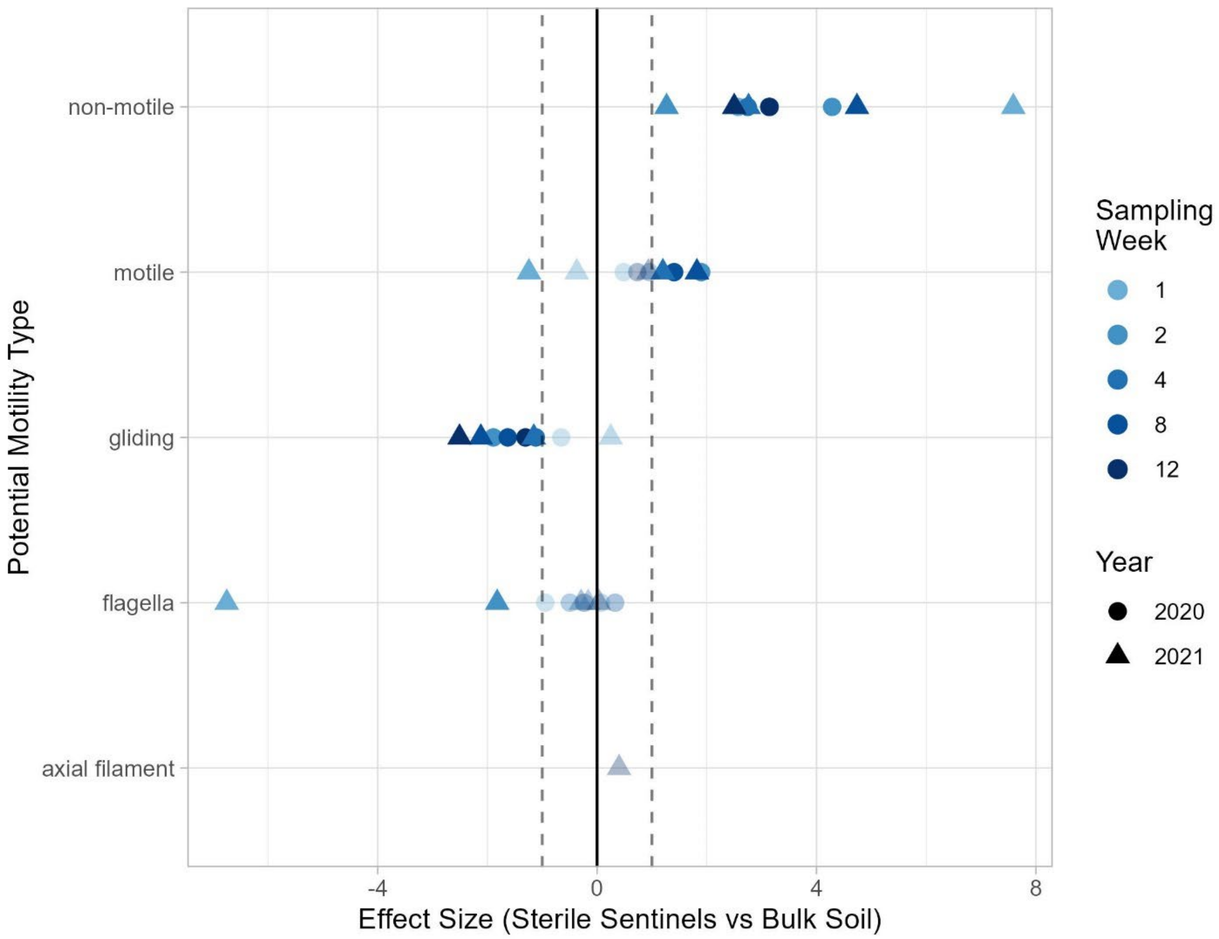

Supplementary Figure 9. Differential abundance of potential motility type in sterile sentinels versus bulk soils. Bacteria with potential gliding motility were more abundant in sentinels. Bacteria predicted as non-motile were less abundant in sterile sentinels. Aldex2 t-tests were performed for each week, so each point represents a single t-test (n = 4). An absolute effect size greater than 1 was considered significant. A negative effect size indicates the trait is more abundant in sterile sentinels relative to bulk soil. Predicted motility was assigned to taxa according to the data compiled by Madin et al. 2020, https://doi.org/10.1038/s41597-020-0497-4.

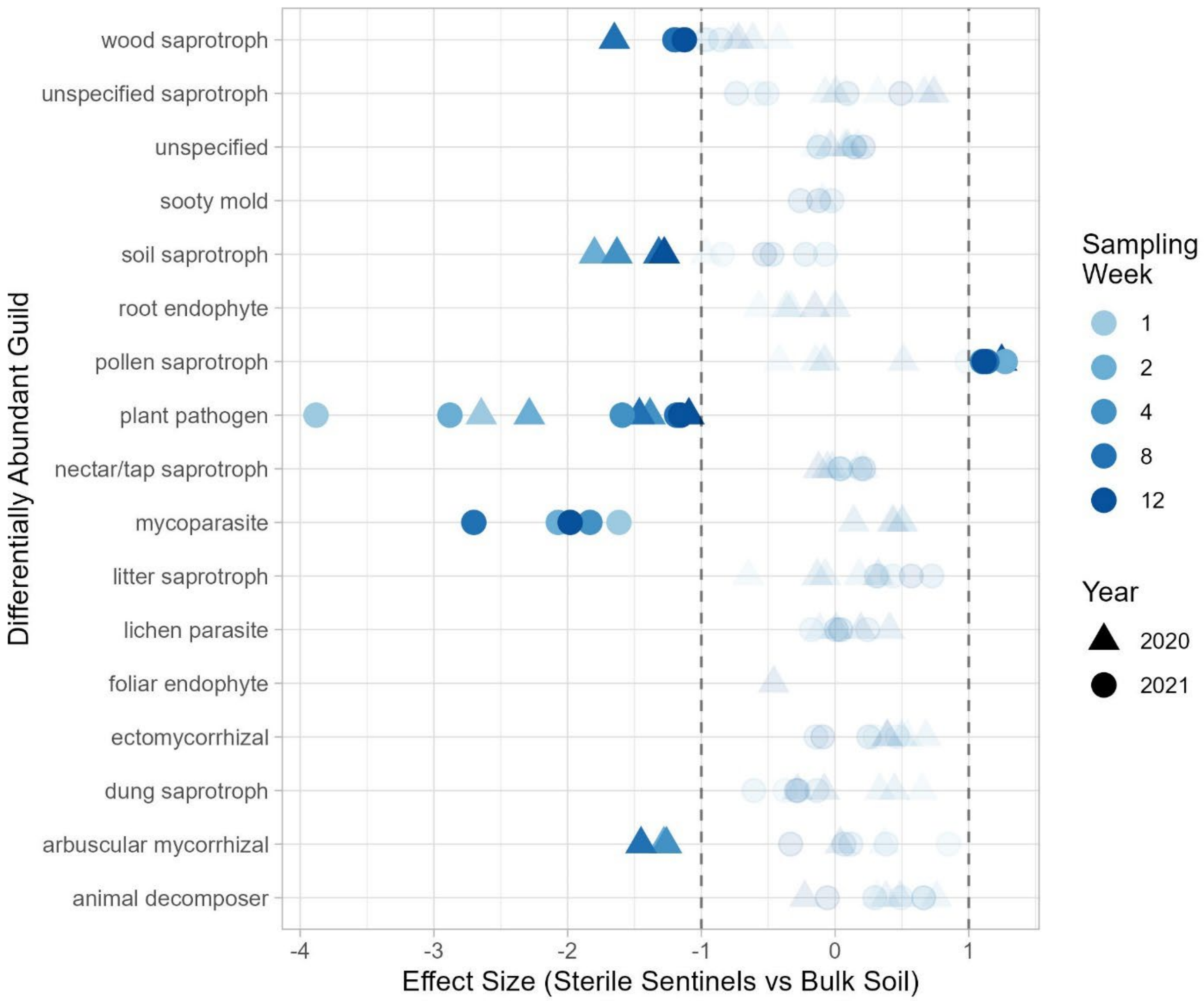

Supplementary Figure 10. Differential abundance of fungal guilds in sterile sentinels versus bulk soil. A negative effect size indicates higher abundance in sentinels, which included plant pathogens and mycoparasites for most weeks tested, while AMF, wood saprotrophs and soil saprotrophs were only more abundant in a few weeks tested. Each point represents a single ALDEx2 t-test (n = 4). Guilds were assigned at the genus level using the FUNguild database (Nguyen et al. 2016).

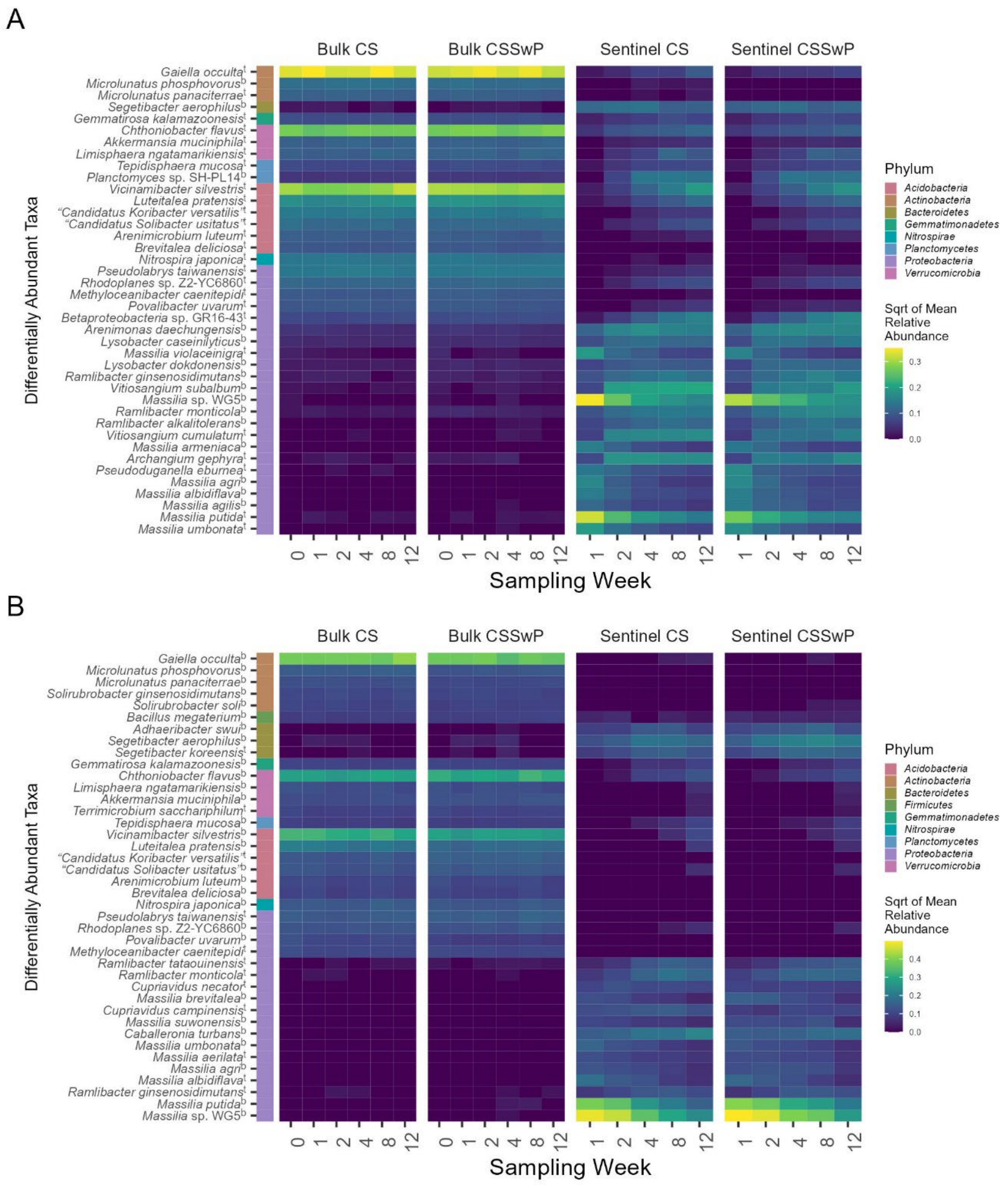

Supplementary Figure 11. Bacterial 2020 (A) and 2021 (B) heatmaps show the square root of taxa mean relative abundance of replicated samples (n = 4). Taxa shown are the 40 most prevalent taxa for each dataset. All taxa are differentially abundant between sample type, rotation, or both. Differential abundance for each test is indicated by superscripts at the end of taxa names. [b] indicates a taxon is differentially abundant for **both** sample type and crop rotations differential abundance tests. [t] indicates differential abundance in the sample **type** test only. [r] indicates differential abundant in crop **rotations** test only. The taxa are ordered by abundance in bulk soil at week 0.

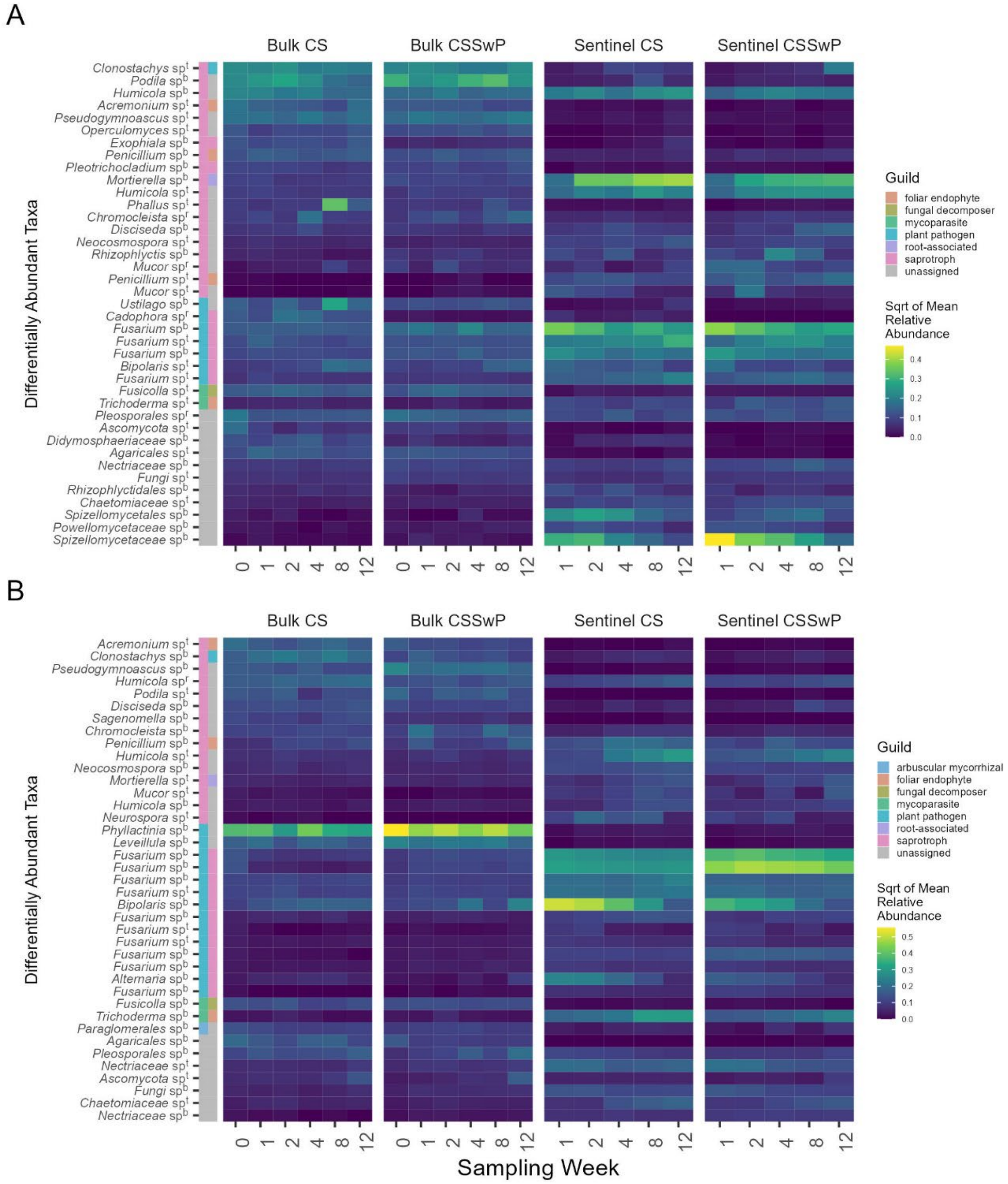

Supplementary Figure 12. Fungal 2020 (A) and 2021 (B) heatmaps show the square root of taxa mean relative abundance for replicated samples (n = 4). The taxa shown are the 40 most prevalent taxa for each dataset. All taxa are differentially abundant between sample type, rotation, or both. Differential abundance for each test is indicated by superscripts at the end of taxa names. [b] indicates a taxon is differentially abundant for **both** sample type and crop rotations differential abundance tests. [t] indicates differential abundance in the sample **type** test only. [r] indicates differential abundant in crop **rotations** test only. The taxa are ordered by abundance in bulk soil at week 0.

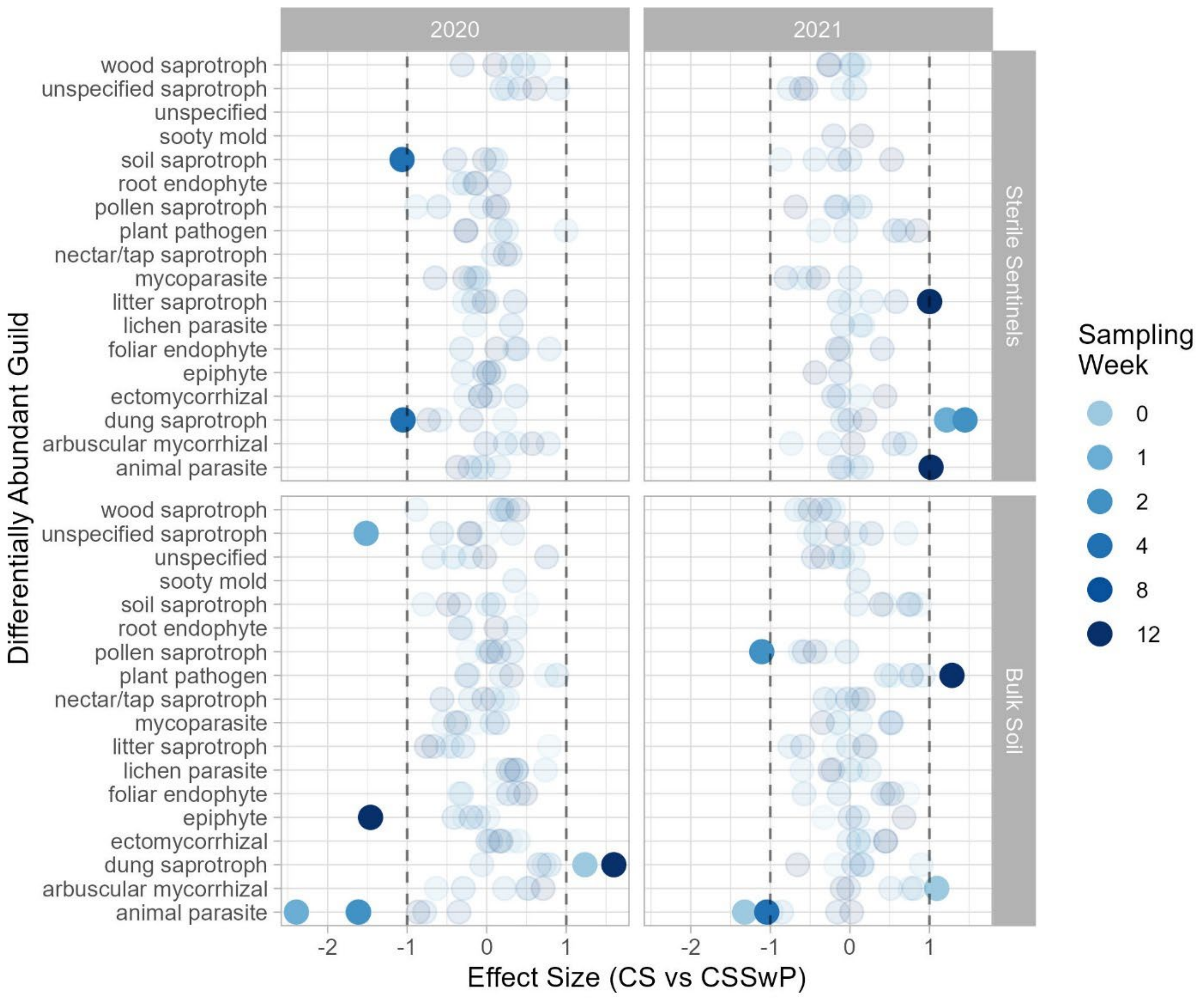

Supplementary Figure 13. Bulk soil and sterile sentinel effect sizes of differential abundance t-tests comparing CS and CSSwP rotations. Few guilds were differentially abundant (effect size > |1|) between rotations for in each sampling week. Negative effect sizes indicate higher relative abundance in CS rotations; positive effect sizes indicate higher relative abundance in CSSwP. Transparent points indicate non-significant differential abundance tests.
